# Sex Hormones, Sex Hormone‐Binding Globulin and Sleep Problems in Females With Polycystic Ovary Syndrome: A Systematic Review and Meta‐Analysis

**DOI:** 10.1111/cen.15219

**Published:** 2025-02-25

**Authors:** Nur K. Abdul Jafar, Meng Fan, Lisa J. Moran, Darren R. Mansfield, Christie J. Bennett

**Affiliations:** ^1^ Monash Centre for Health Research and Implementation, Faculty of Medicine, Nursing and Health Sciences Monash University Clayton Australia; ^2^ Monash Lung and Sleep, Monash Health, Clayton Victoria Australia; ^3^ Faculty of Medicine, Nursing and Health Sciences, School of Psychological Sciences Monash University, Clayton Victoria Australia; ^4^ Be Active Sleep and Eat (BASE) Facility, Department of Nutrition and Dietetics School of Clinical Sciences, Faculty of Medicine, Nursing and Health Sciences, Notting Hill Victoria Australia

**Keywords:** hyperandrogenism, meta‐analysis, obstructive sleep apnea, polycystic ovary syndrome, sex hormones, SHBG, sleep problems

## Abstract

**Objective:**

Sleep problems like obstructive sleep apnea (OSA) are common in polycystic ovary syndrome (PCOS), although the underlying mechanisms are not well understood. We aimed to determine the prevalence of sleep problems, synthesise and appraise studies analysing the associations between serum sex hormones, sex hormone‐binding globulin (SHBG) and sleep problems in females with PCOS.

**Design:**

Systematic review and meta‐analysis.

**Methods:**

A systematic search using MEDLINE, Embase, PsycInfo, CINAHL, Scopus, and Google Scholar was performed till 3 August 2024. Studies that examined serum sex hormones, SHBG or hyperandrogenism with sleep disorders and/or sleep disturbances in PCOS were eligible. Random effects meta‐analyses of sex hormones and SHBG among females with PCOS with compared to without OSA were conducted.

**Results:**

From 4487 screened studies, 24 were included, with nine suitable for meta‐analyses. Among females with PCOS, 46.0% had OSA and 56.0% had other sleep disturbances. SHBG levels were significantly lower in women with PCOS and OSA compared to those without OSA (standardised mean difference = −0.62; 95% CI = −0.82 to −0.42; *I* ^2^ = 0%; 179 participants; *p* < 0.00001), but no differences were seen in total and free testosterone, dehydroepiandrosterone sulfate, androstenedione, and oestradiol. No significant associations between serum sex hormones, SHBG or hyperandrogenism with sleep disturbances in PCOS were observed.

**Conclusion:**

SHBG, rather than hyperandrogenism, may play a more important mechanistic role for OSA in PCOS, while other sleep disturbances exhibit a less severe SHBG profile. These findings enhance comprehension of underlying pathophysiology of sleep problems in PCOS. Further validation across PCOS populations is warranted.

## 1 Introduction

Polycystic ovary syndrome (PCOS) is the most common endocrinological disorder, affecting 8−13% of reproductive‐age women [1] and 3−11% of adolescent girls [2], and is associated with metabolic, reproductive, psychological and dermatological dysfunction [3, 4, 5, 6]. According to the Rotterdam criteria [7], PCOS is diagnosed based on the presence of two out of three features of oligo/amenorrhea, clinical/biochemical hyperandrogenism and polycystic ovary morphology or serum anti‐Mullerian hormone levels [8]. The key pathophysiological features of PCOS include hyperandrogenism, low progesterone and estrogen levels, central adiposity and insulin resistance [9]. Insulin resistance is a prominent feature of PCOS with prevalence rates ranging from 35% to 80% [10]. Intrinsic insulin resistance in women with PCOS is mechanistically distinct from obesity‐associated insulin resistance [11].

Hyperandrogenism can play a role as mechanistic contributor to metabolic disease in PCOS, by promoting central adiposity and further exacerbating insulin resistance [12, 13]. Hyperandrogenism is clinically manifested as hirsutism, acne and alopecia [8, 14], whereas biochemical hyperandrogenism is marked by reduced sex hormone‐binding globulin (SHBG) and increased serum sex hormones or androgens levels such as testosterone, androstenedione and dehydroepiandrosterone sulfate (DHEAS) [15, 16, 17]. SHBG, synthesised in the liver, binds and transports testosterone and other sex steroids in the plasma thus, regulating bioavailable testosterone. The binding of testosterone by SHBG has no biological effects, and only ~1–2% of unbound free testosterone has biological activity [18]. Therefore, SHBG is an effective auxiliary marker for the determination of androgen levels and can be used to estimate the severity of hyperandrogenism [18]. A previous meta‐analysis suggested that women with PCOS and lower SHBG levels, especially for those with high body mass index (BMI), were more likely to have hyperandrogenism, metabolic issues and infertility [19]. Taken together, both sex hormones and SHBG levels should be considered in females with PCOS given their important roles in the development and prognosis of PCOS in clinical practice.

Sleep problems are common in women with PCOS [20], making it important to understand the underlying pathophysiology behind this phenomenon. Androgen excess can promote the development of sleep disorders such as sleep‐disordered breathing (SDB) especially, obstructive sleep apnea (OSA) [20, 21, 22]. OSA is characterised by the repetitive collapse of the upper airway during sleep for at least 10 s, and is associated with oxygen desaturation and/or arousal from sleep [23]. OSA is prevalent above that explained by adiposity and ranges from 17% to 75% in reproductive‐age women with PCOS [24, 25, 26]. Recent studies that examined the proteomic biomarkers of OSA in adults and children have identified an inverse correlation between serum SHBG levels and OSA severity [27, 28]. The authors claim that serum SHBG levels could be a potential indicator to assess the presence and severity of OSA [27, 28].

OSA and PCOS, two common chronic conditions, exhibit similar comorbidities such as insulin resistance, gestational diabetes, type 2 diabetes, hypertension, impaired quality of life, cardiovascular disease and mortality [25, 29]. Beyond OSA, other sleep disorders in PCOS may exist, such as insomnia, circadian rhythm disorders and restless legs syndrome in addition to other sleep disturbances manifesting as daytime sleepiness, poor sleep quality and disrupted sleep‐wake patterns [30, 31, 32, 33, 34, 35, 36, 37, 38]. Together, these sleep problems have shown to impact reproduction function [39], psychological health and lifestyle behaviours [40] in women with PCOS. However, the pathophysiological reason for sleep problems in PCOS is not clear with some, but not all prior research reporting links between hyperandrogenism or SHBG and OSA or other sleep disturbances in PCOS [24, 25, 26, 41].

Therefore, this systematic review and meta‐analysis embodies the first comprehensive evidence synthesis of studies investigating the prevalence of OSA and other sleep disturbances in females with PCOS, and the associations between serum sex hormones, SHBG or hyperandrogenism and sleep problems in females with PCOS.

## 2 Methods

### 2.1 Protocol and Registration

The protocol was registered a priori on the international prospective register of systematic reviews, PROSPERO (CRD42023426688), and reported in accordance with the Preferred Reporting Items for Systematic Reviews and Meta‐Analyses (PRISMA) guidelines [42].

### 2.2 Eligibility Criteria and Search Strategy

The Population‐Exposure‐Outcome (PEO) framework was adopted for this search and eligibility criteria was developed a priori by the study researchers (Table 1) [43]. Studies included were not restricted by language, included females with PCOS of any age, ethnicity, and BMI, and had adequate diagnosis of PCOS (Rotterdam criteria, National Institutes of Health (NIH) definition or Androgen Excess and PCOS Society (AES) definition). Study exposure included clinical/biochemical hyperandrogenism such as hirsutism, serum sex hormones or androgens, SHBG and free androgen index (FAI). Outcome(s) of interest were clinical sleep disorders (e.g., OSA and SDB) or sleep disturbances (including features of a sleep disorder and other non‐clinical sleep measures measured either subjectively (sleep questionnaires) or objectively (polysomnography [PSG] or cardiorespiratory polygraphy). Type of studies that were eligible included observational studies (retrospective, prospective, cross‐sectional, longitudinal, case‐control or cohort studies), randomised controlled trial (RCT) studies, published grey literature and other systematic reviews to identify eligible studies. Studies that investigated females without PCOS or did not report hyperandrogenism or sleep problems were excluded.

**Table 1:** Eligibility criteria (PEO) for study inclusion.

	Population (P)	Exposure (E)	Outcome (O)
Inclusion	Females with PCOS (Clinician‐confirmed PCOS diagnostic criteria e.g., Rotterdam, NIH or AES) of any age, ethnicity, and weight.	Serum sex hormones, hormones relating to hyperandrogenemia or androgens or clinical hyperandrogenism Testosterone, SHBG, FAI, DHEAS, androstenedione, oestradiol and hirsutism by Ferriman‐Gallwey scores.	Diagnosed or screened for sleep disorders or sleep disturbances. Subjective sleep measures that is, Self‐reported sleep questionnaires that measure sleep disturbances or sleep disorders such as daytime sleepiness, risk of sleep apnea, insomnia, sleep quality and restless legs syndrome. Objective sleep measures that is, Sleep apnea on formal sleep studies: Level 1, in‐lab PSG Level 2, PSG set up in home or Level 3, ambulatory limited channel PSG (i.e., home sleep apnea test). Prevalence or severity of OSA (including, sleep apnea, SDB, snoring) by AHI, RDI or ODI. Other sleep disorders such as circadian rhythm (sleep‐wake) disorder or circadian misalignment.
Exclusion	Females without PCOS. Studies with self‐reported PCOS.	Studies not reporting sex hormones/any hormones relating to hyperandrogenemia or androgens or clinical hyperandrogenism.	Studies not reporting sleep disorder or sleep disturbance.

Abbreviations: AES, androgen excess society; AHI, apnea hypopnea index; DHEAS, dehydroepiandrosterone sulphate; FAI, free androgen index; NIH, National institute of health; ODI, oxygen desaturation index; OSA, obstructive sleep apnea; PCOS, polycystic ovary syndrome; PSG, polysomnography; RDI, respiratory distress index; SDB, sleep‐disordered breathing; SHBG, sex hormone binding globulin.

The search terms were based on established terminology used in the PCOS and sleep literature, and consultation were sought from healthcare professionals and clinicians with expertise in PCOS and sleep disorders (Supporting Information S1: 1). To broaden our search further, sex hormone keywords were not included as part of the search strategy, and were used when manually screening full‐texts during the study selection stage. Electronic databases were searched from inception up to 3 August 2024: MEDLINE, Embase, APA PsycInfo (all via Ovid), CINAHL (via EBSCO), Scopus, and Google Scholar (for published grey literature). Only the first 300 search results by article titles from Google Scholar were included for screening in the systematic review as recommended by Haddaway et al. [44].

### 2.3 Study Selection

Screening was undertaken using Covidence (http://www.covidence.org). Duplicates were automatically removed by Covidence or manually removed by two reviewers (N.A.J. and M.F.) using Covidence. Title and abstract screening were conducted in duplicate by two of four independent reviewers (N.A.J., C.B., L.M., and M.F.). The full‐texts were screened in duplicate by two reviewers (N.A.J. and M.F.) and any disagreement was resolved by a third reviewer (C.B.), with discussion among the reviewers to reach consensus, where necessary.

### 2.4 Data Extraction

Data extraction from each full‐text article was completed by one reviewer (N.A.J.) with independent cross‐checking by two reviewers (C.B. and M.F.) to ensure accuracy. Using a standardised data extraction sheet, the following information (if available) was extracted from studies: author, year of publication, country, study design, sample size, population characteristics, setting, age, BMI, method of PCOS diagnosis, prevalence and effect estimates, measurements of sleep and sex hormones, SHBG or hyperandrogenism, and confounders (e.g., age, BMI, or ethnicity). Attempts were made to contact the study co‐authors twice to query for missing or incomplete data [29, 30, 32, 33, 36, 37, 38, 45, 46, 47, 48, 49, 50, 51, 52, 53, 54, 55], units of measurement [46, 51, 56], as well as determine whether cases of OSA were excluded before assessing for sleep disturbances [31, 32, 34, 36, 37, 38, 49], where applicable. Two study co‐authors responded [45, 55], but only one of them had the information requested [55], other study co‐authors did not respond.

### 2.5 Study Quality and Risk of Bias Assessment

Risk of bias of included studies were assessed in duplicate by two independent reviewers (N.A.J. and M.F.) using the Quality Assessment Tool developed by the National Heart, Lung and Blood Institute (NHLBI). Any disagreements were resolved by consensus with a third reviewer (C.B.). We used the NHLBI tools that comprise of 9−14 items for observational cohort and cross‐sectional and case‐control studies [57]. An overall quality score was determined according to the sum of the dichotomised response (yes/no) to each item. Overall quality scores of <40% were considered low quality (rated ‘poor’), scores of 40−60% as moderate quality (rated ‘fair’) and scores > 60% as high quality (rated ‘good’).

### 2.6 Statistical Analysis

The prevalence estimates of OSA and other sleep disturbances in females with PCOS were pooled from the included studies and presented as forest plots of proportions and 95% confidence intervals (CIs) using random‐effects restricted maximum likelihood (REML) models. Data eligible for meta‐analysis were analysed as mean and standard deviation (SD). Median and interquartile (IQR) values were converted to mean (SD), and standard errors (SE) values were converted to SD, in accordance with Cochrane guidelines (http://handbook.cochrane.org/). SHBG and sex hormone concentrations were reported in different units across some studies and thus, converted into the same International System of Units (SI) for meta‐analysis purposes, using online unit conversion tool (http://unitslab.com/node/). Some studies in the meta‐analyses used serum measurements with different hormonal assays to determine SHBG or sex hormones [26, 47, 51, 56, 58, 59]. Therefore, to minimise differences due to assays, standardised mean differences (SMDs) with 95% CIs and inverse‐variance random‐effects model were used for all meta‐analysis [60]. Additionally, two studies that estimated free testosterone by calculation [55, 59], as well as four studies that reported extreme outlier mean values beyond that of women with PCOS [61] for total (120.66−284.77 nmol/L) [46, 56] /free testosterone (156.0−124810 pmol/L) [46, 51, 53], SHBG (0.61−0.93 nmol/L) [51] or DHEAS (6646.6−7270.8 µmol/L) [55] were not included in the respective meta‐analysis. SMDs were interpreted as follows: 0.2 = small effect, 0.5 = moderate effect and 0.8 = large effect [62].

Where only a single study reported a sleep problem, sex hormone or SHBG, or where there was a lack of suitable data for meta‐analysis, these studies were presented descriptively instead. Meta‐analyses and funnel plots were conducted using STATA version 18 software [63] or RevMan Web platform v.7.1.1 (https://revman.cochrane.org/). Statistical heterogeneity between studies were determined by *I* ^2^ statistics in which a value > 50% is considered substantial heterogeneity, implying caution in interpreting the results [64]. Publication bias was assessed by the visual symmetry of funnel plots (≤10 studies) and Egger's regression tests to assess small study effects [65], where applicable. Evidence suggests that potential covariates such as BMI, age, ethnicity and PCOS diagnostic criteria may influence the presence of OSA [41]. Thus, subgroup analyses were conducted to explore the effect of these covariates on the associations between sex hormones and SHBG with OSA, where appropriate. The leave‐one‐out sensitivity analyses were conducted, where each study was sequentially excluded to assess its individual influence on the overall pooled effect estimate. These results were informally compared to each other following Cochrane methods [66].

## 3 Results

### 3.1 Study Characteristics

**Figure 1:**
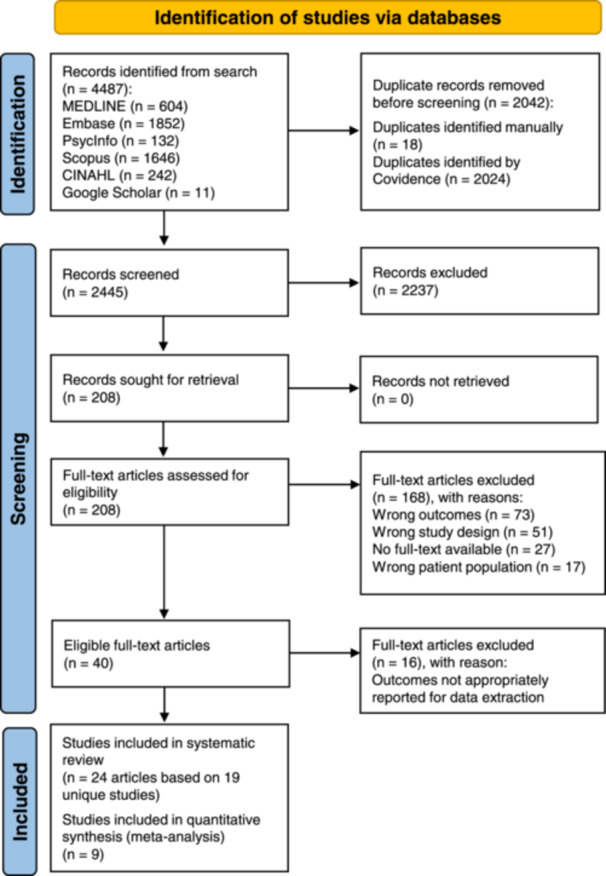
PRISMA flowchart of studies included in the review.

The literature search resulted in 4487 records. After screening 2445 records based on titles and abstracts and 208 full‐texts, 24 articles (corresponding to 19 unique studies) met the eligibility criteria and reported data on the associations between sex hormones, SHBG or hyperandrogenism and sleep problems and thus, included in the review (Figure 1). Of the 24 included studies, only nine studies that examined the associations between serum sex hormones and/or SHBG with OSA [29, 37, 47, 51, 53, 55, 56, 58, 59], had data available for meta‐analysis. Of these nine studies, six studies measured OSA by PSG [29, 47, 51, 56, 58, 59], while three studies determined OSA using BQ [37, 53, 55]. 16 full‐text articles were excluded with reasons (Supporting Information S1: 2). Characteristics of the 24 included studies are presented in Supporting Information S1: 3. 20 studies were cross‐sectional studies [29, 30, 31, 33, 34, 35, 36, 45, 46, 47, 48, 49, 50, 51, 52, 53, 54, 55, 58, 59], three were case‐control studies [32, 37, 38], and one was a prospective cohort study [56]. Total sample sizes ranged from 28 to 505 participants (PCOS: 18 to 328 participants; Non‐PCOS: 18 to 452 participants) [30, 31, 35, 45, 46, 47, 50, 53, 55, 58]. Study populations were mostly Caucasians [30, 32, 34, 45, 46, 47, 49, 50, 55, 59], with six studies from Asian [33, 48, 51, 52, 54, 56] and eight studies from mixed or Turkish ethnicities [29, 31, 35, 36, 37, 38, 53, 58]. PCOS diagnoses for all included studies were confirmed by clinicians. 17 studies diagnosed PCOS using the Rotterdam criteria [29, 31, 32, 33, 34, 36, 37, 38, 48, 51, 52, 53, 54, 55, 56, 58, 59] and seven studies used the NIH criteria [30, 35, 45, 46, 47, 49, 50]. Nineteen studies included adult women with PCOS (mean age range: 20.0−33.5 years) [29, 30, 32, 33, 34, 36, 37, 38, 45, 46, 47, 48, 50, 52, 53, 54, 55, 56, 59], four studies examined adolescent girls with PCOS (mean age range: 15.0−16.9 years) [31, 35, 49, 58], and one was unknown (age not reported) [51]. The mean BMI of participants with PCOS ranged from 21.7 to 45.7 kg/m^2^ [46, 48], with eight out of nine studies included in the meta‐analysis representing overweight/obesity samples [26, 37, 47, 51, 53, 55, 58, 59]. Studies that reported sex hormones, SHBG or hyperandrogenism with sleep problems included total [29, 30, 31, 33, 35, 37, 45, 46, 47, 48, 53, 54, 55, 56, 58, 59], free [30, 31, 45, 46, 47, 51, 52, 53, 55, 58, 59], and bioavailable testosterone [50, 59], DHEAS [29, 47, 51, 55, 59], SHBG [29, 33, 47, 51, 55, 59], androstenedione [29, 48, 54, 55, 59], oestradiol [29, 54, 56], hirsutism [51, 53] and FAI [29, 33, 55]. These studies reported associations with OSA [29, 30, 31, 37, 45, 46, 47, 48, 50, 51, 52, 53, 54, 55, 56, 58, 59], insomnia [33, 37], circadian rhythm disorder [35], daytime sleepiness [31, 34, 37], poor sleep quality [32, 34, 36, 37, 38], restless legs syndrome [37], and/or disrupted sleep‐wake parameters [34, 49].

### 3.2 Quality Appraisal

24 included studies underwent study quality assessment. Seventeen studies had moderate risk of bias and were rated as ‘fair’ quality [26, 31, 33, 34, 35, 37, 45, 47, 48, 49, 51, 52, 53, 54, 56, 58, 59], five studies had high risk of bias and were rated as “poor” quality [30, 32, 36, 38, 46] and two studies had low risk of bias and were rated as ‘good’ quality [50, 55] (Supporting Information S1: 4). The overall average quality rating score for included studies was ‘fair’ (47.0%; range: 33.3−75%). Studies scored well for clearly stating the study objective and specifying study participants (100% out of 24 studies), selecting participants who were representative (87.5% out of 24 studies) and using validated and reliable sleep measurements such as PSG (76% out of 21 cross‐sectional studies). In contrast, studies scored the lowest in measurement of exposure (12.5% out of 24 studies) mainly due to either unspecified hormonal assays [26, 31, 33, 51, 52, 53, 54, 58] or the lack of reliable assays used [30, 32, 34, 36, 38, 45, 46, 47, 48, 49, 56, 59].

### 3.3 Prevalence of Sleep Problems in PCOS

**Figure 2:**
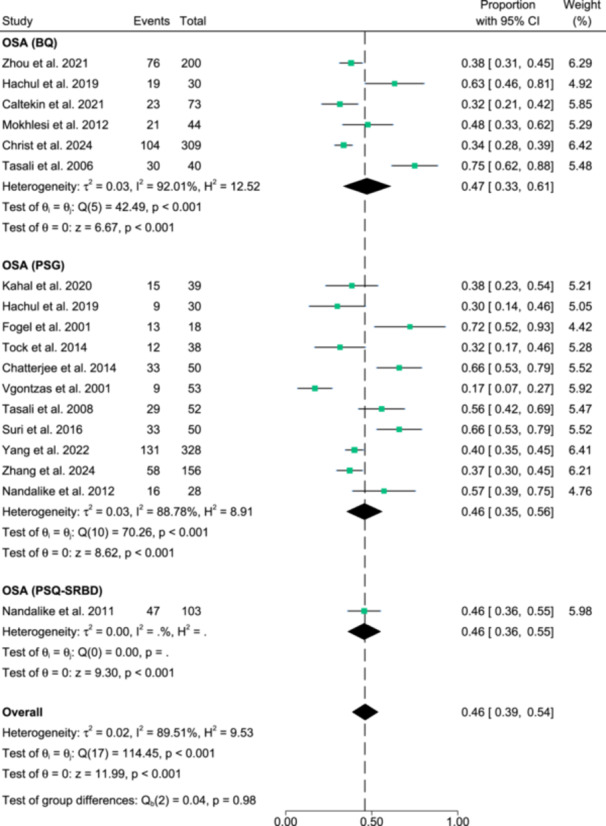
Meta‐analysis depicting the prevalence of Obstructive sleep apnea (OSA) in females with PCOS across Berlin Questionnaire (BQ), polysomnography (PSG) or Paediatric Sleep Questionnaire‐Sleep‐Related Breathing Disorder (PSQ‐SRBD) measurements.

**Figure 3:**
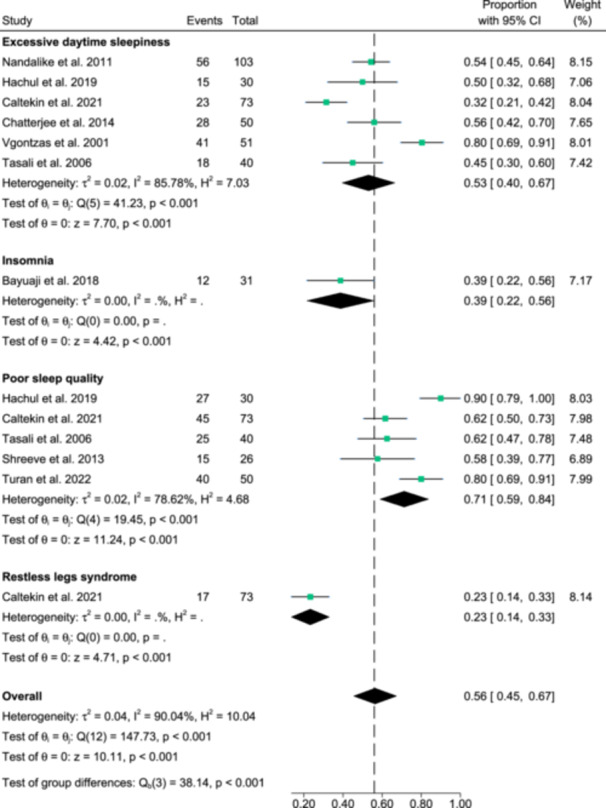
Meta‐analysis depicting the prevalence of other sleep disturbances in females with PCOS.

From Figure 2, the overall prevalence of OSA was 46.0% (95% CI: 39% to 54%) out of 1611 PCOS participants. OSA prevalence rates did not vary significantly across Berlin Questionnaire (BQ), Paediatric Sleep Questionnaire‐Sleep Related Breathing Disorder Subscale (PSQ‐SRBD) and PSG (*p* = 0.98); however, heterogeneity among the studies were significant (*I* ^2^ = 89.5%; *p* < 0.001). Evidence of publication bias was observed from visual asymmetry of the funnel plot and significant Egger's regression test (*p* = 0.02) (Supporting Information S1: 5). From Figure 3, the overall prevalence of other sleep disturbances was 56.0% (95% CI: 45%−67%) out of 454 PCOS participants. The prevalence rates varied significantly across excessive daytime sleepiness, insomnia, poor sleep quality and restless legs syndrome (*p* < 0.001). Heterogeneity among the studies were also significant (*I* ^2^ = 90.04%; *p* < 0.001). Evidence of publication bias was not observed from visual symmetry of the funnel plot and insignificant Egger's regression test (*p* = 0.64) (Supporting Information S1: 5). Sensitivity analyses results altogether showed no differences in significance of the findings (all *p* < 0.001), after exclusion of any individual study from the analysis (data not shown).

### 3.4 Associations Between OSA With Sex Hormones, SHBG or Hyperandrogenism

#### 3.4.1 Meta‐Analysis

**Figure 4:**
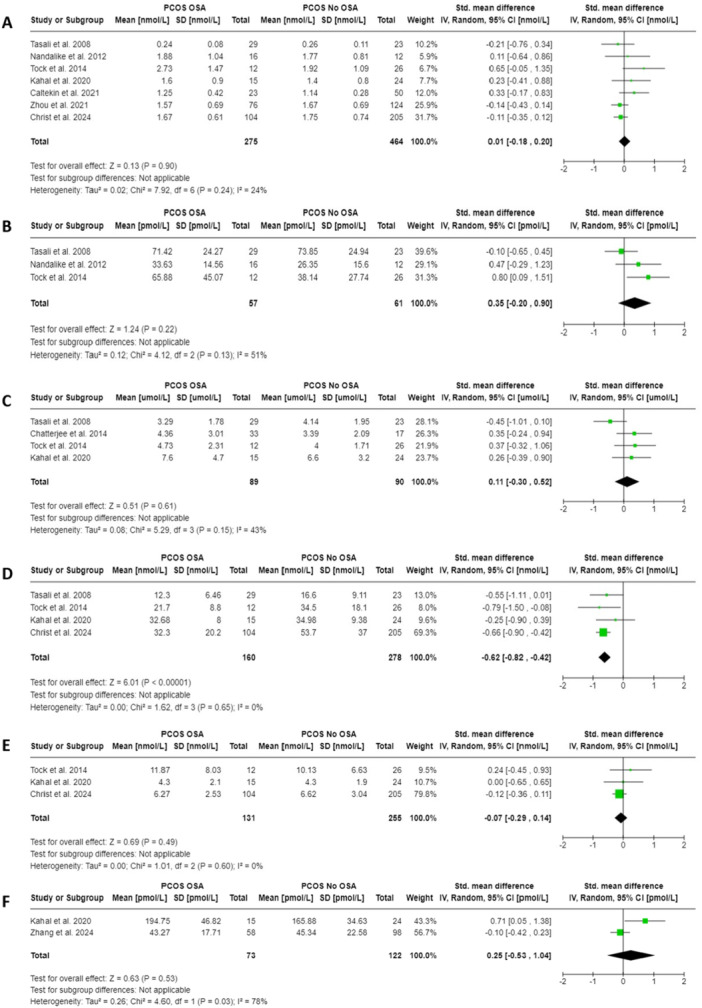
Meta‐analyses depicting the associations between serum sex hormones and SHBG with Obstructive sleep apnea (OSA) in females with PCOS. Forest plot of (A) Total testosterone, (B) Free testosterone, (C) DHEAS, (D) SHBG, (E) Androstenedione and (F) Oestradiol.

Meta‐analysis of seven studies [29, 37, 47, 53, 55, 58, 59] reported overall higher total testosterone levels in women and adolescent girls with PCOS and OSA, compared to those without OSA, but this did not reach statistical significance (SMD = 0.01, 95% CI = −0.18 to 0.20, *I* ^2^ = 24%; *p* = 0.90, 739 participants) (Figure 4A). Three of the seven studies either adjusted or matched their control group for age [29, 47], BMI [29, 47, 58] and/or ethnicity [47]. Meta‐analysis of three studies [47, 58, 59] reported higher free testosterone levels, but not statistically significantly, in women and adolescent girls with PCOS and OSA compared to those without OSA (SMD = 0.35, 95% CI = −0.20 to 0.90, *I* ^2^ = 51%; *p* = 0.22, 118 participants; Figure 4B). Two of the three studies either adjusted or matched their control group for age [47], BMI [47, 58] and/or ethnicity [47]. Meta‐analysis of four studies [29, 47, 51, 59] reported overall higher DHEAS levels, but not significantly, in women with PCOS and OSA, compared to those without OSA (SMD = 0.11, 95% CI = −0.30 to 0.52, *I* ^2^ = 43%; *p* = 0.61, 179 participants; Figure 4C). Meta‐analysis of four studies [29, 47, 55, 59] reported significantly lower SHBG levels in women with PCOS and OSA, compared to those without OSA (SMD = −0.62, 95% CI = −0.82 to −0.42, *I* ^2^ = 0%; *p* < 0.00001, 438 participants; Figure 4D). Meta‐analysis of three studies [29, 55, 59] showed overall androstenedione levels were lower, but not significantly, in women with PCOS and OSA, compared to those without OSA (SMD = −0.07, 95% CI = −0.29 to 0.14, *I* ^2^ = 0%; *p* = 0.49, 386 participants; Figure 4E). Lastly, meta‐analysis of two studies [29, 56] reported overall oestradiol levels were higher, but not significantly, in women with PCOS and OSA, compared to those without OSA (SMD = 0.25, 95% CI = −0.53 to 1.04, *I* ^2^ = 78%; *p* = 0.53, 195 participants; Figure 4F). Visual symmetry inspections of all funnel plots suggest no significant publication bias across the meta‐analyses (Supporting Information S1: 5).

### 3.5 Subgroup and Sensitivity Analysis

Subgroup analyses based on ethnicity depicted positive associations, but not significant, between total testosterone and OSA in Caucasian females with PCOS (SMD = 0.02, 95% CI = −0.38 to 0.43, *p* = 0.91; 3 studies) and in mixed or Turkish females with PCOS (SMD = 0.03, 95% CI = −0.21 to 0.27, *p* = 0.8; 4 studies). An inverse association, albeit not significant, was observed between DHEAS and OSA in Caucasian women with PCOS (SMD = −0.07, 95% CI = −0.87 to 0.74, *p* = 0.87; 2 studies), whereas a positive association though not significant, was observed in mixed ethnicity women with PCOS (SMD = 0.31, 95% CI = −0.13 to 0.74, *p* = 0.17; 2 studies). Subgroup analyses for PCOS diagnostic criteria, BMI, age, and ethnicity (free testosterone, SHBG, androstenedione and oestradiol) were not performed due to the limited number of studies. For sensitivity analyses, the overall effect significance did not change for total testosterone, SHBG, DHEAS and androstenedione, after excluding one study at a time (Supporting Information S1: 6). However, after exclusion of Tasali et al. study [47], the overall effect became significant for free testosterone (SMD = 0.65, 95% CI = 0.13−1.17, *p* = 0.01), whereas for DHEAS, SMD increased modestly from 0.11 to 0.33 and heterogeneity decreased from 43% to 0%. Sensitivity analysis changed the overall effect significance for oestradiol after excluding Zhang et al. study [56] (SMD = 0.71, 95% CI = 0.05−1.38, *p* = 0.04), suggesting that variations in ethnicity between study samples (Mixed [26] vs. Asian [56]) may have influenced the results.

### 3.6 Descriptive Analysis

Those included studies with data not suitable for meta‐analysis reported significant associations between total [45, 48, 54] and free [45, 52, 53, 55, 59] testosterone, SHBG [55], androstenedione [48] and/or oestradiol [54] with OSA, while others did not [30, 31, 37, 46, 50, 51, 53, 55, 56, 59] (Supporting Information S1: 7). One study reported numerically lower, albeit not significant, FAI in women with PCOS and OSA relative to those without OSA [29], while another study reported significantly lower FAI in those with PCOS and low‐risk OSA relative to those with high‐risk OSA [55]. Significant differences in modified Ferriman‐Gallwey Score (mFG) scores for hirsutism were reported in women with PCOS and OSA relative to those without OSA (Mean = 9.82, SD = 2.78 vs. Mean = 8.00, SD = 2.5, *p* = 0.028) [51], but not in another study [53].

### 3.7 Associations Between Other Sleep Problems With Sex Hormones, SHBG or Hyperandrogenism

Two studies reported no significant correlations between testosterone levels and severity of insomnia [33, 37], with one study additionally reported no significant correlations of insomnia with SHBG and FAI in women with PCOS with obesity [33]. One study in adolescent girls with PCOS and obesity [35], reported significant associations between higher free testosterone levels and morning circadian misalignment (*β* = −0.39, 95% CI = −2.12 to −0.37, *p* = 0.006). Seven studies reported no significant associations between serum sex hormones, SHBG or hirsutism with sleep quality, daytime sleepiness, restless legs syndrome or sleep‐wake parameters [31, 32, 34, 36, 37, 38, 49]. The detailed results of these studies are shown in Supporting Information S1: 7.

## 4 Discussion

This systematic review and meta‐analysis show that females with PCOS exhibit high prevalence for OSA (46.0% of 1611 participants) and other sleep disturbances that encompass excessive daytime sleepiness, insomnia, poor sleep quality and restless legs syndrome (56.0% of 454 participants). Moreover, we have observed significantly lower SHBG levels in females with PCOS and OSA compared to those without OSA, but no differences were seen in total and free testosterone, DHEAS, androstenedione, and oestradiol. Overall, there were no significant associations between serum sex hormones, SHBG or hyperandrogenism and other sleep disturbances in PCOS as reported in the literature.

It is known that increased circulating androgen levels can lead to OSA via mechanisms that include increased upper airway collapsibility and impaired sensitivity and/or responsiveness of ventilatory chemoreceptors [67]. In the present meta‐analyses, whilst we observed a trend towards higher serum total and free testosterone, DHEAS and oestradiol levels with OSA in females with PCOS, the pooled associations were small in effect (0.01−0.35) and were not significant. On the other hand, results from SHBG were more consistent, with all four studies showing inverse associations between serum SHBG levels and OSA in females with PCOS [29, 47, 55, 59]. The pooled association result was moderate in effect (−0.62) and extremely significant. This indicates that lower levels of SHBG, rather than hyperandrogenism, may play a more important mechanistic role for OSA in PCOS. It is thought that OSA is linked to hyperandrogenism primarily via modulation of SHBG production in the liver. Insulin resistance and hyperinsulinemia can reduce SHBG, leading to increased risk of OSA [11]. In line with this, lower SHBG levels, but not higher testosterone, have shown to be independently associated with insulin resistance in women with PCOS who are overweight [16]. However, this theory may partly explain the results since one recent study found that only SHBG remained a significant predictor of OSA independent of free testosterone, FAI and multiple markers of insulin resistance in women with PCOS [55]. Another possible theory relates to the impact of OSA on liver function that can decrease SHBG production [68, 69]. The same authors found a negative association between C‐reactive protein (a marker of non‐alcoholic fatty liver disease) and SHBG independent of homoeostatic model assessment for insulin resistance, alcohol consumption, haemoglobin A1C and waist‐hip ratio in women with PCOS [55]. Based on the current evidence, the underlying mechanism for the correlation between low SHBG and OSA in PCOS remains unclear. The cross‐sectional nature of our study precludes investigation of a causal relationship. However, the increased prevalence of OSA could be linked to insulin resistance either directly or indirectly through factors including adiposity, with the potential mechanism of insulin resistance accompanied by hyperinsulinemia leading to low SHBG levels. The status of SHBG as a potential biomarker for OSA in PCOS requires further investigation. More research is needed to examine the pathophysiology and mechanisms explaining the link between OSA and SHBG within the context of PCOS.

The present review extends the literature by synthesising evidence on the prevalence of other sleep problems beyond OSA like excessive daytime sleepiness, insomnia, poor sleep quality and restless legs syndrome in females with PCOS. We observed that the most frequently assessed and reported sleep disturbance domains in females with PCOS were poor sleep quality (71.0%) and excessive daytime sleepiness (53.0%). In fact, sleep disturbances have shown to be more prevalent amongst women with PCOS even after adjusting for BMI, depressive symptoms, demographic and comorbid factors [70]. However, it remains unclear if OSA symptoms might have contributed to the underlying sleep disturbances. Except for one study that ruled out OSA diagnosis (where none of the adolescent girls had AHI ≥ 5) [49], the rest of the included studies did not specifically mention exclusion of OSA from their analysis. This could likely due to the high costs associated with evaluating all participants suspected of having OSA using laboratory PSG. We also observed in the descriptive analysis, no significant associations between sex hormones, SHBG or hyperandrogenism with sleep quality, daytime sleepiness, restless legs syndrome, and sleep‐wake patterns in PCOS [31, 32, 36, 37, 38, 49]. These findings imply that females with PCOS who experience sleep disturbances may exhibit a less severe (or subclinical) hyperandrogenism or SHBG profile. Thus, understanding the variations in the underlying pathophysiology based on clinical severity is useful to target screening and management of sleep problems in PCOS.

To our knowledge, this systematic review and meta‐analysis is the first comprehensive evidence of observational studies investigating the associations between serum sex hormones, SHBG or hyperandrogenism across various domains of sleep problems in PCOS. One of the strengths of the present meta‐analysis includes studies representing diverse ethnic groups from United States of America, Turkey, South America and Asia. This may also partly explain the reason for the high variability in prevalence rates observed for OSA and other sleep problems among the studies. Ethnic variations in craniofacial anatomy, fat distribution and low arousal thresholds can differentially affect the risk of OSA [71]. Studies included in the meta‐analyses were rated as moderate to high quality, indicating sound validity in the evidence synthesis and findings. Subgroup analysis by ethnicity for total testosterone and DHEAS was conducted showing no change in the significance of the pooled results. Sensitivity analyses was also performed showing no change in the overall significance of the meta‐analysis results for total testosterone, SHBG, DHEAS and androstenedione, after excluding one study at a time.

This review has also some limitations. Several studies did not have suitable data for inclusion in the meta‐analysis and did not account for important confounder factors such as age, BMI and ethnicity. Further, insulin resistance, as a common mechanism linking PCOS and sleep problems, could be a key driver of changes in SHBG levels, but it was not explicitly analysed as a confounder with SHBG in previous studies [29, 47, 55, 59]. Moreover, the research lacks data on other types of populations for example, Asians and adolescent girls, limiting generalisation of findings to the global PCOS population. Due to the limited studies, subgroup analyses were not conducted to test the impact of the aforementioned factors on the associations between sex hormones and SHBG with OSA in PCOS. Studies included in this meta‐analysis have shown considerable variability with regard to androgens and OSA risk. This may be attributed to underpowered analyses, variability of measurement methods for SHBG and androgens, definitions of sleep problems and variability in diagnostic criteria for PCOS. Findings from this meta‐analysis should therefore be interpreted with caution as heterogeneity across studies may compromise the comparability of the results. Until then, large‐scale observational studies in the general PCOS population is warranted using gold‐standard androgens and sleep assessments.

## 5 Conclusion

Sleep problems are highly common in females with PCOS, making it important to understand the underlying pathophysiology behind this phenomenon. A significant inverse association between serum SHBG levels and OSA in PCOS is consistently reported. It can be inferred that SHBG, rather than hyperandrogenism, plays a more important mechanistic role for OSA in PCOS, while other sleep disturbances exhibit a less severe SHBG profile. SHBG could likely be a predictor of OSA in PCOS, but further validation of findings across other ethnicities, lean, and adolescent PCOS populations is warranted.

## Conflicts of Interest

The authors declare no conflicts of interest.

## Supporting information

Supporting information.

## Data Availability

The data sets generated during and/or analysed during the current study are not publicly available, but are available from the corresponding author on reasonable request.
