# Supplementary material for: Sex Hormones, Sex Hormone‐Binding Globulin and Sleep Problems in Females With Polycystic Ovary Syndrome: A Systematic Review and Meta‐Analysis
Source: Clin Endocrinol (Oxf). 2025 Feb 25;102(6):708–20. doi: 10.1111/cen.15219 (PMC12046544; doi:10.1111/cen.15219)
Supplement: Supplementary file 1 — Supporting information. [file CEN-102-708-s001.docx]

**Supporting Information**

**Supporting Information 1**

***Search strategies for electronic database searches***

**Ovid MEDLINE(R) All, Embase Classic + Embase:**

| **#** | **Query** |
| --- | --- |
| 1 | exp Polycystic Ovary Syndrome/ |
| 2 | "Polycystic Ovar*".tw. |
| 3 | PCOS.tw. |
| 4 | PCOD.tw. |
| 5 | (sclerocystic adj3 ovar*).tw. |
| 6 | "stein leventhal".tw. |
| 7 | 1 or 2 or 3 or 4 or 5 or 6 |
| 8 | exp Sleep/ |
| 9 | exp sleep disordered breathing/ |
| 10 | exp insomnia/ |
| 11 | exp hypersomnia/ |
| 12 | exp excessive daytime sleepiness/ |
| 13 | exp restless legs syndrome/ |
| 14 | exp Sleep Disorders, Circadian Rhythm/ |
| 15 | Sleep*.tw. |
| 16 | OSA*.tw. |
| 17 | OSAHS.tw. |
| 18 | SAS.tw. |
| 19 | SAHS.tw. |
| 20 | hypopnea*.tw. |
| 21 | hypopnoea*.tw. |
| 22 | [insomnia.tw](http://insomnia.tw/). |
| 23 | [hypersomnia.tw](http://hypersomnia.tw/). |
| 24 | EDS.tw. |
| 25 | restless legs [syndrome.tw](http://syndrome.tw/). |
| 26 | RLS.tw. |
| 27 | [circadian.tw](http://circadian.tw/). |
| 28 | Polysomnograph*.tw. |
| 29 | Actigraph*.tw. |
| 30 | PSQ.tw. |
| 31 | Berlin Questionnaire.tw. |
| 32 | SDQ.tw. |
| 33 | STOP Questionnaire.tw. |
| 34 | ESS.tw. |
| 35 | AIS.tw. |
| 36 | ISI.tw. |
| 37 | PIRS.tw. |
| 38 | PSQI.tw. |
| 39 | Jenkins Questionnaire.tw. |
| 40 | 8 or 9 or 10 or 11 or 12 or 13 or 14 or 15 or 16 or 17 or 18 or 19 or 20 or 21 or 22 or 23 or 24 or 25 or 26 or 27 or 28 or 29 or 30 or 31 or 32 or 33 or 34 or 35 or 36 or 37 or 38 or 39 |
| 41 | 7 and 40 |

**Ovid APA PsycInfo:**

| **#** | **Query** |
| --- | --- |
| 1 | exp endocrine sexual disorders/ |
| 2 | "Polycystic Ovar*".tw. |
| 3 | PCO*.tw. |
| 4 | (sclerocystic adj3 ovar*).tw. |
| 5 | "stein leventhal".tw. |
| 6 | 1 or 2 or 3 or 4 or 5 |
| 7 | exp Sleep/ |
| 8 | exp Sleep Apnea/ |
| 9 | exp Insomnia/ |
| 10 | exp Hypersomnia/ |
| 11 | exp Restless Leg Syndrome/ |
| 12 | exp Sleep Wake Disorders/ |
| 13 | Sleep*.tw. |
| 14 | OSA*.tw. |
| 15 | OSAHS.tw. |
| 16 | SAS.tw. |
| 17 | SAHS.tw. |
| 18 | hypopnea*.tw. |
| 19 | hypopnoea*.tw. |
| 20 | [insomnia.tw](http://insomnia.tw/). |
| 21 | [hypersomnia.tw](http://hypersomnia.tw/). |
| 22 | EDS.tw. |
| 23 | restless legs [syndrome.tw](http://syndrome.tw/). |
| 24 | RLS.tw. |
| 25 | [circadian.tw](http://circadian.tw/). |
| 26 | Polysomnograph*.tw. |
| 27 | Actigraph*.tw. |
| 28 | PSQ.tw. |
| 29 | Berlin Questionnaire.tw. |
| 30 | SDQ.tw. |
| 31 | STOP Questionnaire.tw. |
| 32 | ESS.tw. |
| 33 | AIS.tw. |
| 34 | ISI.tw. |
| 35 | PIRS.tw. |
| 36 | PSQI.tw. |
| 37 | Jenkins Questionnaire.tw. |
| 38 | 7 or 8 or 9 or 10 or 11 or 12 or 13 or 14 or 15 or 16 or 17 or 18 or 19 or 20 or 21 or 22 or 23 or 24 or 25 or 26 or 27 or 28 or 29 or 30 or 31 or 32 or 33 or 34 or 35 or 36 or 37 |
| 39 | 6 and 38 |

**EBSCO CINAHL:**

| **#** | **Query** |
| --- | --- |
| S1 | MH "Polycystic Ovary Syndrome" |
| S2 | (polycystic N3 ovar*) or PCOS or "stein leventhal" or (sclerocystic N3 ovar*) |
| S3 | S1 OR S2 |
| S4 | MH "Sleep Apnea Syndromes+" |
| S5 | MH "Sleep Disorders, Circadian Rhythm+" |
| S6 | sleep* |
| S7 | osa or sas or osahs or sahs or hypopnea* or hypopnoea* |
| S8 | insomnia or hypersomnia or eds |
| S9 | "restless legs syndrome" or rls |
| S10 | circadian |
| S11 | polysomnograph* |
| S12 | actigraph* |
| S13 | psq |
| S14 | "berlin questionnaire" or bq |
| S15 | sdq |
| S16 | "STOP questionnaire" |
| S17 | ess |
| S18 | ais |
| S19 | isi |
| S20 | pirs |
| S21 | psqi |
| S22 | "jenkins questionnaire" |
| S23 | S4 OR S5 OR S6 OR S7 OR S8 OR S9 OR S10 OR S11 OR S12 OR S13 OR S14 OR S15 OR S16 OR S17 OR S18 OR S19 OR S20 OR S21 OR S22 |
| S24 | S3 AND S23 |

**Scopus search string:**

( TITLE-ABS-KEY ( sclerocystic W/3 ovar* ) OR TITLE-ABS-KEY ( polycystic W/3 ovar* ) OR TITLE-ABS-KEY ( "polycystic ovary syndrome" OR "ovary polycystic disease*" OR pcos OR "stein leventhal" ) AND TITLE-ABS-KEY ( sleep* ) OR TITLE-ABS-KEY ( osa OR sas OR osahs OR sahs OR hypopnea* OR hypopnoea* ) OR TITLE-ABS-KEY ( insomnia OR hypersomnia OR eds ) OR TITLE-ABS-KEY ( "restless legs syndrome" OR rls ) OR TITLE-ABS-KEY ( circadian ) OR TITLE-ABS-KEY ( polysomnograph* ) OR TITLE-ABS-KEY ( actigraph* ) OR TITLE-ABS-KEY ( psq ) OR TITLE-ABS-KEY ( berlin AND questionnaire OR bq ) OR TITLE-ABS-KEY ( sdq ) OR TITLE-ABS-KEY ( stop AND questionnaire ) OR TITLE-ABS-KEY ( ess ) OR TITLE-ABS-KEY ( ais ) OR TITLE-ABS-KEY ( isi ) OR TITLE-ABS-KEY ( pirs ) OR TITLE-ABS-KEY ( psqi ) OR TITLE-ABS-KEY ( jenkins AND questionnaire ) )

**Supporting Information 2**

***List of eligible full-text studies excluded with reasons (n = 16)***

| **#** | **Citation** | **Reason** |
| --- | --- | --- |
| 1 | Underland LJ, Agarwal C, Sin S, Punjabi N, Heptulla R, Arens R. Insulin sensitivity and obstructive sleep apnea in adolescents with polycystic ovary syndrome. Minerva Endocrinology. 2022 Apr 7. | Did not report the association between sex hormones and Obstructive sleep apnea. |
| 2 | Gholizadeh‐Moghaddam M, Ghasemi‐Tehrani H, Askari G, Jaripur M, Clark CC, Rouhani MH. Effect of magnesium supplementation in improving hyperandrogenism, hirsutism, and sleep quality in women with polycystic ovary syndrome: A randomized, placebo‐controlled clinical trial. Health Science Reports. 2023 Jan;6(1):e1013. | Did not report the association between sex hormones or hyperandrogenism and sleep quality. |
| 3 | Alizadeh M, Karandish M, Asghari Jafarabadi M, Heidari L, Nikbakht R, Babaahmadi Rezaei H, Mousavi R. Metabolic and hormonal effects of melatonin and/or magnesium supplementation in women with polycystic ovary syndrome: A randomized, double-blind, placebo-controlled trial. Nutrition & Metabolism. 2021 Dec;18(1):1-1. | Did not report the association between sex hormones and sleep quality. |
| 4 | Zeng X, Ye J, Yan X, Zhang J, Guo J, Tao X, Zheng X, Tong M, Huang Y, Zhang M, He C. Staying up late increases cardiovascular disease risk in women with polycystic ovary syndrome. Human Reproduction. 2023 Jun 5. | Did not report the association between sex hormones and sleep disturbances. |
| 5 | Oberg E, Blomberg L, Åkerstedt T, Hirschberg AL. Different sleep pattern in over-weight/obese women with polycystic ovary syndrome. Frontiers in Endocrinology. 2023 Feb 10. | Did not report the association between sex hormones and sleep variables. |
| 6 | Li H, Liu M, Zhang C. Women with polycystic ovary syndrome (PCOS) have reduced melatonin concentrations in their follicles and have mild sleep disturbances. BMC Women's Health. 2022 Mar 21;22(1):79. | Did not report the association between sex hormones and sleep variables. |
| 7 | Eisenberg E, Legro RS, Diamond MP, Huang H, O’Brien LM, Smith YR, Coutifaris C, Hansen KR, Santoro N, Zhang H. Sleep habits of women with infertility. The Journal of Clinical Endocrinology & Metabolism. 2021 Nov 1. | Did not report the association between sex hormones and sleep variables. |
| 8 | Torres-Zegarra C, Sundararajan D, Benson J, Seagle H, Witten M, Walders-Abramson N, Simon SL, Huguelet P, Nokoff NJ, Cree-Green M. Care for adolescents with polycystic ovary syndrome: development and prescribing patterns of a multidisciplinary clinic. Journal of Pediatric and Adolescent Gynecology. 2021 Oct 1;34(5):617-25. | Did not report the association between sex hormones and sleep variables. |
| 9 | Yang Y, Deng H, Li T, Xia M, Liu C, Bu XQ, Li H, Fu LJ, Zhong ZH. The mental health of Chinese women with polycystic ovary syndrome is related to sleep disorders, not disease status. Journal of Affective Disorders. 2021 Mar 1;282:51-7. | Did not report the association between sex hormones and sleep disorders. |
| 10 | Simon S, Rahat H, Carreau AM, Garcia-Reyes Y, Halbower A, Pyle L, Nadeau KJ, Cree-Green M. Poor sleep is related to metabolic syndrome severity in adolescents with PCOS and obesity. The Journal of Clinical Endocrinology & Metabolism. 2020 Apr;105(4):e1827-34. | Did not report the association between sex hormones and sleep variables. |
| 11 | de Sousa G, Schlüter B, Menke T, Trowitzsch E, Andler W, Reinehr T. Longitudinal analyses of polysomnographic variables, serum androgens, and parameters of glucose metabolism in obese adolescents with polycystic ovarian syndrome. Sleep and Breathing. 2012 Dec;16:1139-46. | Did not report the association between sex hormones and sleep variables. |
| 12 | de Sousa G, Schlüter B, Buschatz D, Menke T, Trowitzsch E, Andler W, Reinehr T. The impact of insulin resistance and hyperandrogenemia on polysomnographic variables in obese adolescents with polycystic ovarian syndrome. Sleep and Breathing. 2012 Mar;16:169-75. | Did not report the association between sex hormones and sleep variables. |
| 13 | Vgontzas AN, Trakada G, Bixler EO, Lin HM, Pejovic S, Zoumakis E, Chrousos GP, Legro RS. Plasma interleukin 6 levels are elevated in polycystic ovary syndrome independently of obesity or sleep apnea. Metabolism. 2006 Aug 1;55(8):1076-82. | Did not report the association between sex hormones and sleep variables. |
| 14 | Kangwolkij N, Sophonsritsuk A, Tantrakul V, Charakorn C, Tantanavipas S. Prevalence and Predictive Factors for Being High-risk of Obstructive Sleep Apnea Using Berlin Questionnaire in Polycystic Ovary Syndrome: Age-and BMI-matched study. Thai Journal of Obstetrics and Gynaecology. 2022 Nov 1:413-22. | Did not report the association between sex hormones and sleep variables. |
| 15 | Albogami SS, Albassam WB, Alghamdi EG, Alabdullatif A, Alajlan ZA, AlAwad SI, Hamd ZY. Prevalence of polycystic ovary syndrome by ultrasound and it's relation with endometrial hyperplasic and depression. Journal of Radiation Research and Applied Sciences. 2023 Sep 1;16(3):100637. | Did not report the association between sex hormones and sleep variables. |
| 16 | Karjula S, Morin-Papunen L, Franks S, Auvinen J, Järvelin MR, Tapanainen JS, Jokelainen J, Miettunen J, Piltonen TT. Population-based data at ages 31 and 46 show decreased HRQoL and life satisfaction in women with PCOS symptoms. The Journal of Clinical Endocrinology & Metabolism. 2020 Jun;105(6):1814-26. | Did not report the association between sex hormones and sleep variables. |

**Supporting Information 3**

| ***Characteristics of included studies in the systematic review (n = 24)*** | | | | | | | | | | | | |
| --- | --- | --- | --- | --- | --- | --- | --- | --- | --- | --- | --- | --- |
| **Author,**  **year, country** | **Population, Setting** | **Ethnicity** | **Study design** | **Study groups** | **Females with PCOS^a^** | | | | **Sex hormone measurement** | **Sleep problem measurement** | **Summary of findings** | **Quality rating^b^** |
|  |  |  |  |  | **PCOS criteria** | **Subgroup (n)** | **Age (y)** | **BMI (kg/m^2^)** |  |  |  |  |
| Fogel et al., 2001, USA | Adults, Community (controls) Hospital (cases) | Caucasian | Cross-sectional | PCOS (n = 18)  Age- and weight-matched Non-PCOS (n = 18) | NIH | NA | 31.1 (1.3) | 36.9 (1.3) | Total T and DHEAS, immunoassay kit;  free T, radioimmunoassay | Severity of OSA (AHI) by PSG | AHI correlated with serum total T (r = 0.52, *P* < .001) and Free T (r = 0.50, *P* < .05) in women with PCOS. | Fair |
| Vgontzas et al., 2001, USA | Adults,  General public (controls)  Sleep laboratory (cases) | Caucasian | Cross-sectional | PCOS (n = 53)  Non-PCOS (n = 452) | NIH | SDB: 9  No SDB: 44 | All: 30.4 (0.9)  SDB: 34.0 (2.8)  No SDB: 29.6 (0.8) | All: 38.7 (1.1) SDB: 45.7 (2.6)  No SDB: 37.2 (1.1) | Total T, immunoassay kit;  free T, radioimmunoassay | Presence of SDB (AHI≥10 + clinical symptomatology) by PSG | No significant differences in total T or free T between PCOS (SDB vs. No SDB). | Poor |
| Tasali et al., 2006, USA | Adults, University endocrinology clinics (cohorts 1 and 2), Sleep laboratory (cohort 2) | Caucasian | Cross-sectional (Two cohorts) | Cohort 1 PCOS assessed with sleep surveys (n = 40)  Cohort 2 PCOS assessed with sleep study (n = 8) | NIH | NA | Cohort 1: 31.4 (1.2)  Cohort 2: 31.0 (2.1) | Cohort 1: 39.8 (1.3)  Cohort 2: 41.1 (2.9) | Total T, immunoassay kit; free T, competitive protein-binding assay | Cohort 1: subjective sleep quality, PSQI; daytime sleepiness, ESS; risk of sleep apnea, BQ  Cohort 2: Severity of SDB (AHI, ODI) by PSG | No significant correlations between androgen levels and severity of SDB in women with PCOS. | Poor |
| Tasali et al., 2008, USA | Adults Community (controls)  University endocrinology clinics (cases) | Caucasian | Cross-sectional  (from a cohort study) | PCOS (n = 52)  Non-PCOS (n = 21) | NIH | OSA: 29  No OSA: 23 | All: 29.7 (0.7)  OSA: 31.6 (1.0)  No OSA: 27.3 (0.7) | All: 39.2 (1.0), 23.2-58.8  OSA: 42.2 (1.1)  No OSA: 35.3 (1.4) | Total T, immunoassay kit; free T and SHBG, competitive protein-binding assay; DHEAS, radioimmunoassay | Presence and severity of OSA (AHI≥5) by PSG | After controlling for age, BMI, ethnicity no significant association between AHI and free T, and no significant difference in  total T, free T, SHBG and DHEAS levels between PCOS (OSA vs. No OSA). | Fair |
| Yang et al., 2009, Taiwan | Adults, Community (control)  Obstetrics and gynaecology clinic in hospital (cases) | Asian | Cross-sectional | PCOS (n = 18)  Age- and BMI-matched Non-PCOS (n = 10) | Rotterdam | NA | 29.1 (1.43) | 21.7 (0.57) | Total T and androstenedione, radioimmunoassay; SHBG, measurement not reported | Severity of SDB (AHI_NREM_) by PSG | AHI_NREM_ correlated with T (B = 2.039, *P* = .032) and androstenedione (B = -1.036, *P* = .006), but not with SHBG. | Fair |
| de Sousa et al., 2011, Germany | Adolescents, Outpatient obesity and endocrine department hospital | Caucasian | Cross-sectional  (from a cohort study) | PCOS (n = 31)  Non-PCOS (n = 19) | NIH | OSA: 0  No OSA: 31 | 15.0 (1.0) | 32.7 (6.2) | Total T, DHEAS and androstenedione, chemiluminescence immunoassay | Sleep apnea/quality parameters by PSG | No significant correlations between PSG sleep-related variables and androgen levels. | Fair |
| Nandalike et al., 2011, USA | Adolescents,  Electronic medical information database (Clinical Looking Glass, CLG) | Mixed  (African American, Hispanic, White) | Cross-sectional | PCOS (n = 103)  Age-, race-, BMI Z-score matched Non-PCOS (n = 90) | Rotterdam | SDB: 47  No SDB: 56  EDS: 56  No EDS: 47 | All: 16.9 (1.5)  SDB:16.8 (1.5)  No SDB: 16.9 (1.5)  EDS: 16.8 (1.5)  No EDS: (16.9 (1.6) | BMI Z-score  All: 1.9 (0.6)  SDB: 2.1 (0.5)  No SDB: 1.7 (0.6)  EDS: 2 (0.6)  No EDS: 1.7 (0.6) | Total T and free T, extracted from EPF (not specified) | Presence of SDB, PSQ-SRDB;  Daytime sleepiness, ESS | T levels similar between girls with PCOS (SDB vs. No SDB or EDS vs. No EDS) even after controlling for age, BMI and ethnicity. | Fair |
| Nandalike et al., 2012, USA | Adolescents,  Sleep-disordered database (controls)  Electronic medical information database (Clinical Looking Glass, CLG) (cases) | Mixed  (African American, Hispanic, White) | Cross-sectional | PCOS (n = 28)  Age-, race-, BMI Z-score matched Non-PCOS (n = 28)  BMI Z-score-matched males (n = 28) | Rotterdam | OSA: 16  No OSA: 12 | All: 16.8 (1.9)  OSA: 16.8 (2.1)  No OSA: 16.6 (1.7) | BMI Z-score  All: 2.4 (0.4)  OSA: 2.5 (0.3)  No OSA: 2.3 (0.5) | Total T and free T, extracted from EPF (not specified) | Presence of OSA (AHI>5 or apnea index >1) by PSG | Testosterone levels similar between obese girls with PCOS (OSA vs. No OSA). | Fair |
| Mokhlesi et al., 2012, USA | Adults,  Community (controls)  Endocrinology clinics and community (cases) | Caucasian | Cross-sectional | PCOS (n = 44)  Non-PCOS (n = 34) | NIH | High risk OSA:21  Low risk OSA: 23 | 27 (5.0) | 35.1 (11.4) | Total T, LC-MS; bioavailable T, binding of T to SHBG (radioimmunoassay) and albumin (spectrophotometry) | OSA by BQ | After controlling for BMI,  bioavailable T was not an independent predictor of OSA risk in women with PCOS. | Good |
| Shreeve et al., 2013, UK | Adults, Gynaecology outpatient clinic in hospital | Caucasian | Case-control | PCOS (n = 26)  Non-PCOS (n = 26) | Rotterdam | Poor sleep quality: 15  Normal sleep quality: 11 | 29.8 (3.7) | 29.3 (8.2) | T, FAI, SHBG and oestradiol, measurement not reported | Sleep quality by PSQI | No significant correlations between sleep quality and hormone profiles in women with PCOS. | Poor |
| Chatterjee et al., 2014, India | Adults, Gynaecology outpatient department and endocrinology clinic in hospital | Asian | Cross-sectional | PCOS (n = 50) | Rotterdam | SDB: 33  No SDB: 17 | Not reported | SDB: 29.8 (3.4)  No SDB: 24.36 (2.29) | Free T, DHEAS and SHBG, hormonal assay (not specified); hirsutism from FG score | Presence and severity of SDB (RDI≥5 + clinical symptoms or RDI>15) by PSG | No significant differences in free T, DHEAS, and SHBG between PCOS (SDB vs. No SDB). Significant difference in FG scores between PCOS (SDB vs. No SDB, *P* = .028). After controlling for BMI, no significant correlation between free T and RDI. | Fair |
| Tock et al., 2014, Brazil | Adults, Endocrinology division in university | Caucasian | Cross-sectional | PCOS (n = 38) | Rotterdam | OSA: 12  No OSA: 26 | All: 28.3 (6.8)  OSA: 28.3 (5.0)  No OSA: 28.4 (7.5) | All: 32.9 (7.7)  OSA: 37.8 (4.8)  No OSA: 30.67 (7.7) | Total T and DHEAS, chemiluminescence immunoassay; Androstenedione and SHBG, immunoassay system; Serum free T and bioavailable T, estimated by Vermeulen equation | Presence and severity of OSA (AHI≥5) by PSG | After adjusting for BMI, hyperandrogenemia (free T >= 1.07 ng/dL) associated with OSA in women with PCOS (8.2 fold). SHBG lower in PCOS (OSA vs. No OSA, *P* = .027). Free T higher in PCOS (OSA vs. no OSA, *P* = .014). No significant difference between OSA vs. no OSA for Androstenedione, DHEAS, total and bioavailable T. | Fair |
| Suri et al., 2016, India | Adults, Gynaecology outpatient department and endocrinology clinic in hospital | Asian | Cross-sectional | PCOS (n = 50)  Age-matched women Non-PCOS (n = 100) | Rotterdam | SDB: 33  No SDB: 17 | 27.9 (6.44) | 28.0 (4.01) | Free T, DHEAS, hormonal assay (not specified) | SDB by BQ; Severity of SDB (RDI≥5 + clinical symptoms or RDI>15) by PSG | Free T significantly correlated with RDI values (r = 0.377, *P* = .007). | Fair |
| Bayuaji et al., 2018, Indonesia | Adults, Assisted reproductive or fertility clinic in hospital | Asian | Cross-sectional | PCOS (n = 31) | Rotterdam | Insomnia: 12  No insomnia: 19 | 30.19 (3.67) | 31.21 (6.17) | Total T, SHBG; measurement not reported; FAI | Insomnia by AIS | No significant correlations between total T, SHBG and FAI with AIS score. | Fair |
| Hachul et al., 2019, Brazil | Adults, Endocrinology division in university | Caucasian | Cross-sectional | PCOS (n = 30)  Non-PCOS (n = 14) | Rotterdam | NA | 29.7 (1.2) | 34.3 (1.1) | Total T, immunoassay; Serum free T and bioavailable T, estimated by Vermeulen equation | Presence of OSA (AHI≥5 + clinical symptoms), PSG; Other sleep parameters, PSG; Daytime sleepiness, ESS; Sleep quality, PSQI | After controlling for age, no differences in ESS, PSQI scores, PSG sleep-related parameters and AHI events in PCOS (hyperandrogenism vs. no hyperandrogenism). | Fair |
| Simon et al., 2019, USA | Adolescents, Specialty clinics in hospital | Mixed  (White, Black, Hispanic) | Cross-sectional | PCOS (n = 59)  Non-PCOS (n = 33) | NIH | NA | 15.7 (1.8) | BMI percentile:  97.4 (2.3) | Total T, HPLC-MS; free T, equilibrium dialysis; SHBG, chemiluminescent immunoassay | Circadian misalignment, radioimmunoassay (dim-light melatonin); Daytime sleepiness (CASQ and SDSC) | After controlling for daytime sleepiness and SDB, later melatonin offset after wake time  associated with higher serum free T levels regardless of PCOS. | Fair |
| Kahal et al., 2020, UK | Adults, PCOS, weight management and endocrinology clinics in hospital | Mixed  (White, Asian) | Cross-sectional | PCOS (n = 39) | Rotterdam | OSA: 15  No OSA: 24 | All: 32.2 (8.9)  OSA: 33 (26-43)  No OSA: 29.5 (27-33) | All: 34.1 (7.9)  OSA: 37.3 (7.3)  No OSA: 32.2 (7.8) | T, SHBG, FAI, DHEAS, androstenedione and oestradiol, measurements not reported | Presence of OSA, BQ + ESS follow-up with home-based portable sleep monitor (ODI≥5) | After controlling for age and BMI, no significant differences in androgen levels in PCOS (OSA vs. No OSA). | Fair |
| Zhou et al., 2021, USA | Adults, PCOS clinic in university | Mixed  (White, Asian, Hispanic, Black, Native American) | Cross-sectional | PCOS (n = 200) | Rotterdam | High-risk OSA: 76  Low-risk OSA: 124 | All: 28.0 (6.2)  High-risk OSA: 28.4 (6.5)  Low-risk OSA: 27.8 (6.0) | All: 30.9 (9.0)  High-risk OSA: 38.1 (8.2)  Low-risk OSA: 26.5 (6.3) | Total T, free T, FG scores, DHEAS and androstenedione, measurement not reported | OSA by BQ | T levels and FG scores similar between groups, but those high risk for OSA had higher levels of free T compared with low risk OSA (*P* < .001). | Fair |
| Karasu et al., 2021, Turkey | Adults, Gynaecology outpatient clinic at university | Mixed (European-Asian) | Cross-sectional | PCOS (n = 111)  Non-PCOS (n = 108) | Rotterdam | NA | 25.1 (5.8) | 26.5 (5.1) | Free T, immunoassay; hirsutism from FG score; DHEAS, oestradiol, measurements not reported | Sleep quality by PSQI | No significant correlations between free T, modified FG scores and PSQI score among women with PCOS. | Poor |
| Caltekin et al., 2021, Turkey | Adults, Gynaecology and neurology outpatient clinics at hospital | Turkish | Case-control | PCOS (n = 73)  Non-PCOS (n = 63) | Rotterdam | High-risk OSA: 23  Low-risk OSA: 50  Poor sleep quality:45  Normal sleep quality: 28  EDS: 23  No EDS: 50  RLS:17  No RLS: 56 | 26.03 (5.02) | 25.9 (4.37) | Total T, measurement not reported | Sleep quality, PSQI; Daytime sleepiness, ESS; insomnia, ISI; Risk of OSA, BQ; RLS, IRLSSG criteria | No significant correlation between T levels and PSQI, ESS and ISI in PCOS group. No significant difference in T levels in PCOS (low-risk OSA vs. high risk OSA; RLS vs No RLS). | Fair |
| Yang Rui et al., 2022, China | Adults (infertile), University hospital | Asian | Cross-sectional | PCOS (n = 328) | Rotterdam | OSA: 131  No OSA: 197 | No OSA: 28.8 (3.2)  Mild OSA: 29.2 (3.9)  Moderate OSA: 30 (4)  Severe OSA: 33.5 (3.6) | No OSA: 24.2 (3.6)  Mild OSA: 28.4 (3.7)  Moderate OSA: 31.7 (4.9)  Severe OSA: 32.1 (3.3) | T, androstenedione, oestradiol, measurements not reported | Presence and severity of OSA (AHI≥5) by type III portable sleep monitor | T levels associated with OSA (OR = 1.11, *P* < .05). No difference in T and androstenedione levels between OSA vs No OSA. Significant difference mild OSA vs. No OSA for oestradiol (*P* < .05). | Fair |
| Turan et al., 2022, Turkey | Adults, Gynaecology and obstetrics outpatient clinic | Turkish | Case-control | PCOS (n = 50)  Non-PCOS (n = 51) | Rotterdam | Poor sleep quality: 40  Normal sleep quality: 10 | All: 20 (18.8-23.2)  Poor sleep quality: 20 (19-24.7)  Normal sleep quality: 19.5 (18-21.2) | All: 24.3 (20.5-27)  Poor sleep quality: 24.3 (20.2-26.9)  Normal sleep quality: 23.1 (20.4-28.2) | Total T and DHEAS, immunoassay | Sleep quality by PSQI | After controlling for age and BMI, T and DHEAS levels were not significantly associated with sleep quality in women with PCOS. | Poor |
| Zhang et al., 2024, China | Adults underwent IVF aged 20-35, University hospital | Asian | Prospective cohort | PCOS (n = 156) | Rotterdam | OSA: 58  No OSA: 98 | All: 30.1 (3.5)  OSA: 30.8 (3.5)  No OSA: 29.7 (3.5) | All: 23.9 (3.6)  OSA: 25.4 (3.5)  No OSA: 23 (3.3) | T, oestradiol, and progesterone Chemiluminescence immunoassay | Presence of OSA (AHI≥5), portable non-contact radar sleep monitor | No significant differences in T, oestradiol and progesterone in PCOS (OSA vs. No OSA). | Fair |
| Christ et al., 2024, USA | Adults,  University-based multidisciplinary PCOS clinic | Caucasian | Cross-sectional | PCOS (n = 309) | Rotterdam | High-risk OSA: 104  Low-risk OSA: 205 | High-risk OSA: 28.7 (5.9)  Low-risk OSA: 28.0 (5.2) | High-risk OSA: 38.0 (9.1)  Low-risk OSA: 27.2 (7.0) | Total T and androstenedione, LC/MS-MS; DHEAS and SHBG, Electrochemiluminescence immunoassay; Free T, Direct analog enzyme immunoassay and by calculation | OSA by BQ | Higher free T and FAI and lower SHBG (all *P* < .001) (High-risk vs. Low-risk). After adjusted for WHR and all reported variables,  only SHBG remained significant in the multivariable model (B = -0.02 (0.01), *P* = .02). | Good |
| Abbreviations: AHI, apnea-hypopnea index; AIS, Athens insomnia scale; BMI, body mass index; BQ, Berlin questionnaire; CASQ, Cleveland adolescent sleepiness questionnaire; DHEAS, Dehydroepiandrosterone sulfate; EDS, Excessive daytime sleepiness; EPF, electronic patient file; ESS, Epworth sleepiness scale; FAI, free androgen index; FG, Ferriman-Gallwey; HPLC-MS, High-performance liquid chromatography and mass spectrometry; IRLSS, International restless legs syndrome study group; ISI, Insomnia severity index; LC/MS-MS, Liquid chromatography with tandem mass spectrometry; NIH, National institute of health; ODI, Oxygen desaturation index; OSA, obstructive sleep apnea; PCOS, polycystic ovary syndrome; PSG, polysomnography; PSQI, Pittsburgh sleep quality index; PSQ-SRDB, Paediatric sleep questionnaire sleep-related disordered breathing; NA, not applicable; RDI, Respiratory disturbance index; SDB, Sleep disordered breathing; SDSC, Sleep disturbances scale for children; SHBG, sex hormone binding globulin; T, testosterone; WHR, waist-hip ratio.  ^a^ Values are present as mean (SD) and/or range unless otherwise stated.  ^b^ Quality score rated based on the National Heart, Lung, and Blood Institute (NHLBI) quality assessment tool. | | | | | | | | | | | | |

**Supporting Information 4**

***Quality appraisals for observational cohort and cross-sectional studies using the NHLBI study quality assessment tool***

| **Author, year, country** | **Q1** | **Q2** | **Q3** | **Q4** | **Q5** | **Q6** | **Q7** | **Q8** | **Q9** | **Q10** | **Q11** | **Q12** | **Q13** | **Q14** | **Quality rating and % score^a^** |
| --- | --- | --- | --- | --- | --- | --- | --- | --- | --- | --- | --- | --- | --- | --- | --- |
| Fogel et al., 2001, USA | Yes | Yes | Yes | Yes | No | Not applicable | Not applicable | No | No | No | Yes | Yes | Not reported | Yes | Fair (58.3%) |
| Vgontzas et al., 2001, USA | Yes | Yes | No | No | No | Not applicable | Not applicable | No | No | No | Yes | No | Yes | No | Poor (33.3%) |
| Tasali et al., 2006, USA | Yes | Yes | No | Yes | No | Not applicable | Not applicable | No | No | No | No | Not reported | Yes | No | Poor (33.3%) |
| Tasali et al., 2008, USA | Yes | Yes | Not reported | Yes | No | Not applicable | Not applicable | No | No | No | Yes | Not reported | Yes | Yes | Fair (50%) |
| Yang et al., 2009, Taiwan | Yes | Yes | Not reported | Yes | No | Not applicable | Not applicable | No | No | No | Yes | Not reported | Yes | Yes | Fair (50%) |
| De Sousa et al., 2011, Germany | Yes | Yes | Not reported | Yes | No | Not applicable | Not applicable | No | No | No | Yes | Not reported | Yes | No | Fair (41.7%) |
| Nandalike et al., 2011, USA | Yes | Yes | Yes | Yes | No | Not applicable | Not applicable | No | Not reported | No | Yes | No | Yes | Yes | Fair (58.3%) |
| Nandalike et al., 2012, USA | Yes | Yes | Yes | No | No | Not applicable | Not applicable | No | Not reported | No | Yes | Not reported | Yes | Yes | Fair (50%) |
| Mokhlesi et al., 2012, USA | Yes | Yes | Yes | Yes | Yes | Not applicable | Not applicable | No | Yes | No | Yes | Not reported | Yes | Yes | Good (75%) |
| Chatterjee et al., 2014, India | Yes | Yes | Not reported | Yes | No | Not applicable | Not applicable | No | Not reported | No | Yes | Not reported | Yes | Yes | Fair (50%) |
| Tock et al., 2014, Brazil | Yes | Yes | Not reported | Yes | No | Not applicable | Not applicable | No | No | No | Yes | Not reported | Yes | Yes | Fair (50%) |
| Suri et al., 2016, India | Yes | Yes | Yes | Yes | No | Not applicable | Not applicable | No | Not reported | No | Yes | Not reported | Yes | Yes | Fair (58.3%) |
| Bayuaji et al., 2018, Indonesia | Yes | Yes | Not reported | Yes | No | Not applicable | Not applicable | No | Not reported | No | Yes | Not reported | Yes | No | Fair (41.7%) |
| Hachul et al., 2019, Brazil | Yes | Yes | Not reported | Yes | No | Not applicable | Not applicable | No | No | No | Yes | Not reported | No | Yes | Fair (41.7%) |
| Simon et al., 2019, USA | Yes | Yes | Not reported | Yes | No | Not applicable | Not applicable | No | Yes | No | No | Not reported | Yes | No | Fair (41.7%) |
| Kahal et al., 2020, UK | Yes | Yes | Not reported | Yes | Yes | Not applicable | Not applicable | No | Not reported | No | No | Yes | Yes | Yes | Fair (58.3%) |
| Zhou et al., 2021, USA | Yes | Yes | Not reported | Yes | No | Not applicable | Not applicable | No | Not reported | No | Yes | Not reported | Yes | No | Fair (41.7%) |
| Karasu et al., 2021, Turkey | Yes | Yes | Not reported | Yes | No | Not applicable | Not applicable | No | No | No | Yes | Not reported | Not reported | No | Poor (33.3%) |
| Yang Rui et al., 2022, China | Yes | Yes | Not reported | Yes | Yes | Not applicable | Not applicable | No | Not reported | No | No | Not reported | Yes | No | Fair (41.7%) |
| Zhang et al., 2024, China | Yes | Yes | Not reported | Yes | No | Not applicable | Not applicable | No | No | No | No | Yes | Yes | No | Fair (41.7%) |
| Christ Jacob et al., 2024, USA | Yes | Yes | Yes | Yes | No | Not applicable | Not applicable | No | Yes | No | Yes | Not reported | Yes | Yes | Good (66.7%) |

Abbreviations: NHLBI, National Heart, Lung, and Blood Institute.

^a^ Overall quality score was calculated based on the sum of the dichotomized response (yes/no) to each item. “Not applicable” items were not included in the calculation. Overall quality scores of <40% were considered low quality (rated “poor”), scores of 40-60% as moderate quality (rated “fair”) and scores >60% as high quality (rated “good”).

Q1: Was the research question or objective in this paper clearly stated?

Q2: Was the study population clearly specified and defined?

Q3: Was the participation rate of eligible persons at least 50%?

Q4: Were all the subjects selected or recruited from the same or similar populations (including the same time period)? Were inclusion and exclusion criteria for being in the study prespecified and applied uniformly to all participants?

Q5: Was a sample size justification, power description, or variance and effect estimates provided?

Q6: For the analyses in this paper, were the exposure(s) of interest measured prior to the outcome(s) being measured?

Q7: Was the timeframe sufficient so that one could reasonably expect to see an association between exposure and outcome if it existed?

Q8: For exposures that can vary in amount or level, did the study examine different levels of the exposure as related to the outcome (e.g., categories of exposure, or exposure measured as continuous variable)?

Q9: Were the exposure measures (independent variables) clearly defined, valid, reliable, and implemented consistently across all study participants?

Q10: Was the exposure(s) assessed more than once over time?

Q11: Were the outcome measures (dependent variables) clearly defined, valid, reliable, and implemented consistently across all study participants?

Q12: Were the outcome assessors blinded to the exposure status of participants?

Q13: Was loss to follow-up after baseline 20% or less?

Q14: Were key potential confounding variables measured and adjusted statistically for their impact on the relationship between exposure(s) and outcome(s)?

***Quality appraisals for case-control studies using the NHLBI study quality assessment tool***

| **Author, year, country** | **Q1** | **Q2** | **Q3** | **Q4** | **Q5** | **Q6** | **Q7** | **Q8** | **Q9** | **Q10** | **Q11** | **Q12** | **Quality rating and % score^a^** |
| --- | --- | --- | --- | --- | --- | --- | --- | --- | --- | --- | --- | --- | --- |
| Shreeve et al., 2013, UK | Yes | Yes | No | Yes | No | Yes | Not reported | No | No | No | Not reported | No | Poor (33.3%) |
| Caltekin et al., 2021, Turkey | Yes | Yes | No | Yes | Yes | Yes | Not reported | No | No | No | Not reported | Not applicable | Fair (45.5%) |
| Turan et al., 2022, Turkey | Yes | Yes | No | No | No | Yes | Not reported | No | No | No | Not reported | Yes | Poor (33.3%) |

Abbreviations: NHLBI, National Heart, Lung, and Blood Institute.

^a^ Overall quality score was calculated based on the sum of the dichotomized response (yes/no) to each item. “Not applicable” items were not included in the calculation. Overall quality scores of <40% were considered low quality (rated “poor”), scores of 40-60% as moderate quality (rated “fair”) and scores >60% as high quality (rated “good”).

Q1: Was the research question or objective in this paper clearly stated and appropriate?

Q2: Was the study population clearly specified and defined?

Q3: Did the authors include a sample size justification?

Q4: Were controls selected or recruited from the same or similar population that gave rise to the cases (including the same timeframe)?

Q5: Were the definitions, inclusion and exclusion criteria, algorithms or processes used to identify or select cases and controls valid, reliable, and implemented consistently across all study participants?

Q6: Were the cases clearly defined and differentiated from controls?

Q7: If less than 100 percent of eligible cases and/or controls were selected for the study, were the cases and/or controls randomly selected from those eligible?

Q8: Was there use of concurrent controls?

Q9: Were the investigators able to confirm that the exposure/risk occurred prior to the development of the condition or event that defined a participant as a case?

Q10: Were the measures of exposure/risk clearly defined, valid, reliable, and implemented consistently (including the same time period) across all study participants?

Q11: Were the assessors of exposure/risk blinded to the case or control status of participants?

Q12: Were key potential confounding variables measured and adjusted statistically in the analyses? If matching was used, did the investigators account for matching during study analysis?

**Supporting Information 5**

***Funnel plots of publication bias***

**1. Meta-analysis of the prevalence of OSA in PCOS**


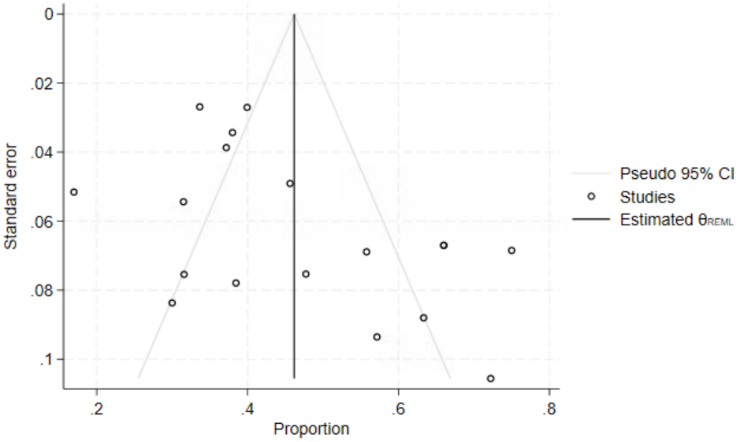


Visual asymmetry; Eggers test for publication bias: Z = 2.29; P = 0.02 – observed small study effects.

**2. Meta-analysis of the prevalence of other sleep disorders/disturbances in PCOS**


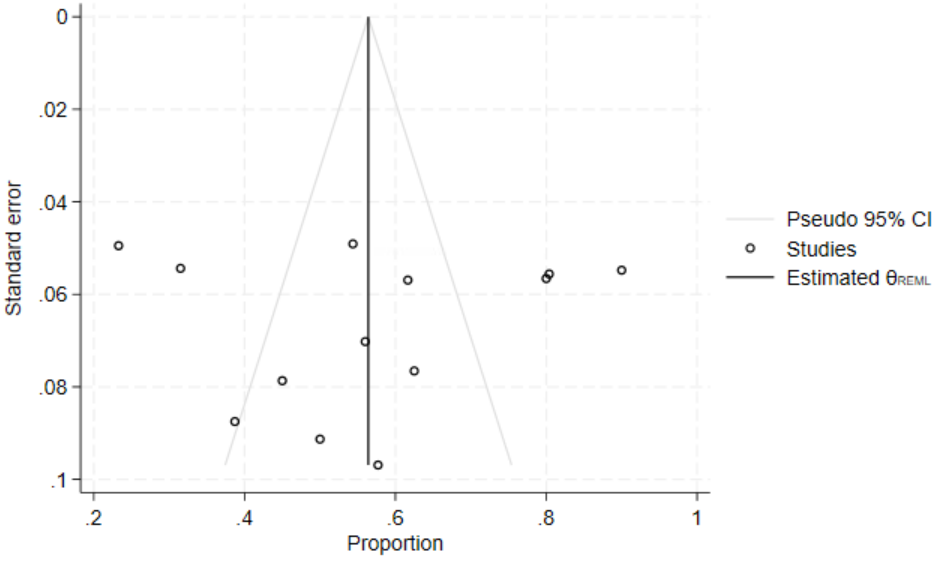


Visual symmetry; Eggers test for publication bias: Z = -0.47; P = 0.64 – no observed small study effects.

**3. Meta-analysis of the association between total testosterone and OSA in PCOS**


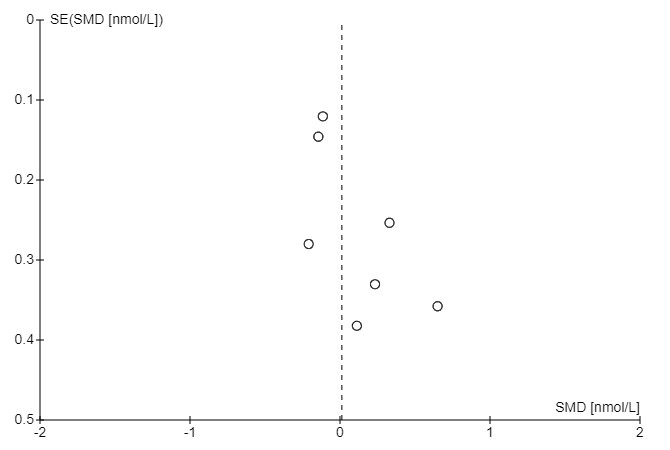


Visual symmetry – no observed small study effects.

**4. Meta-analysis of the association between free testosterone and OSA in PCOS**


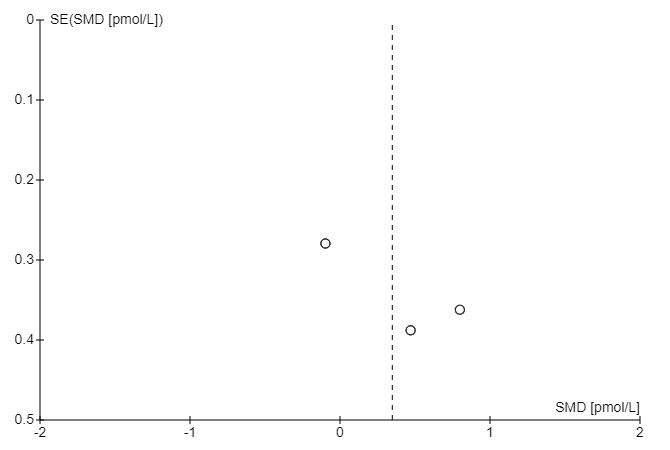


Visual symmetry – no observed small study effects.

**5. Meta-analysis of the association between DHEAS and OSA in PCOS**


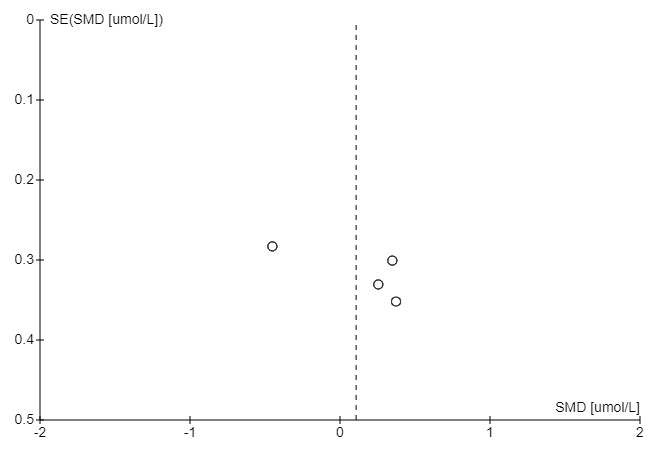


Visual symmetry – no observed small study effects.

**6. Meta-analysis of the association between SHBG and OSA in PCOS**


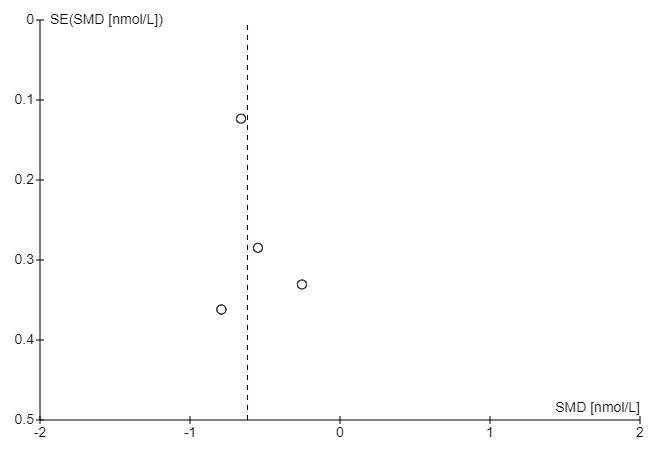


Visual symmetry – no observed small study effects.

**7. Meta-analysis of the association between Androstenedione and OSA in PCOS**


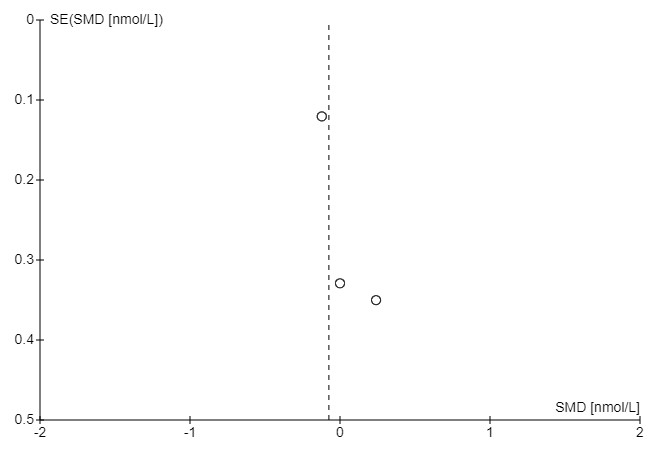


Visual symmetry – no observed small study effects.

**8. Meta-analysis of the association between Oestradiol and OSA in PCOS**


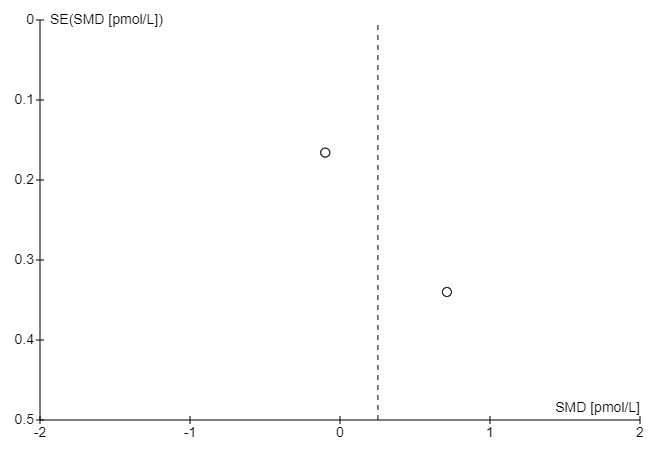


Visual symmetry – no observed small study effects.

**Supporting Information 6**

| ***Sensitivity analyses*** | | | | | | | | |
| --- | --- | --- | --- | --- | --- | --- | --- | --- |
| **Study excluded** | **OSA measurement** | ***N*** | **SMD** | **95% CI** | **Test for overall effect** | | **Heterogeneity** | |
|  |  |  |  |  | **Z** | ***P* value** | ***P* value** | ***I*^2^ (%)** |
| *Total testosterone* | | | | | | | | |
| Tasali et al. 2008 | PSG | 687 | 0.05 | -0.17 to 0.27 | 0.47 | 0.64 | 0.19 | 33 |
| Nandalike et al. 2012 | PSG | 711 | 0.02 | -0.20 to 0.23 | 0.18 | 0.86 | 0.17 | 36 |
| Tock et al. 2014 | PSG | 701 | -0.06 | -0.21 to 0.10 | 0.73 | 0.46 | 0.52 | 0 |
| Kahal et al. 2020 | PSG | 700 | 0 | -0.21 to 0.21 | 0.01 | 0.99 | 0.20 | 31 |
| Caltekin et al. 2021 | PSG | 666 | -0.05 | -0.23 to 0.14 | 0.48 | 0.63 | 0.33 | 13 |
| Zhou et al. 2021 | BQ | 539 | 0.08 | -0.16 to 0.33 | 0.67 | 0.50 | 0.22 | 28 |
| Christ et al. 2024 | BQ | 430 | 0.09 | -0.17 to 0.34 | 0.67 | 0.50 | 0.22 | 28 |
| *Free testosterone* | | | | | | | | |
| Tasali et al. 2008 | PSG | 66 | 0.65 | 0.13 to 1.17 | 2.44 | 0.01* | 0.54 | 0 |
| Nandalike et al. 2012 | PSG | 90 | 0.32 | -0.56 to 1.20 | 0.72 | 0.47 | 0.05 | 74 |
| Tock et al. 2014 | PSG | 80 | 0.12 | -0.42 to 0.67 | 0.44 | 0.66 | 0.23 | 29 |
| *DHEAS* | | | | | | | | |
| Tasali et al. 2008 | PSG | 127 | 0.33 | -0.04 to 0.69 | 1.73 | 0.08 | 0.97 | 0 |
| Chatterjee et al. 2014 | PSG | 129 | 0.03 | -0.50 to 0.56 | 0.11 | 0.92 | 0.12 | 53 |
| Tock et al. 2014 | PSG | 141 | 0.04 | -0.48 to 0.55 | 0.14 | 0.89 | 0.11 | 55 |
| Kahal et al. 2020 | PSG | 140 | 0.07 | -0.48 to 0.62 | 0.25 | 0.81 | 0.08 | 60 |
| *SHBG* | | | | | | | | |
| Tasali et al. 2008 | PSG | 386 | -0.63 | -0.84 to -0.41 | 5.70 | <0.00001* | 0.46 | 0 |
| Tock et al. 2014 | PSG | 400 | -0.60 | -0.81 to -0.39 | 5.62 | <0.00001* | 0.50 | 0 |
| Kahal et al. 2020 | PSG | 399 | -0.66 | -0.87 to -0.44 | 6.07 | <0.00001* | 0.87 | 0 |
| Christ et al. 2024 | BQ | 129 | -0.52 | -0.88 to -0.16 | 2.80 | 0.005* | 0.54 | 0 |
| *Androstenedione* | | | | | | | | |
| Tock et al. 2014 | PSG | 348 | -0.11 | -0.33 to 0.11 | 0.95 | 0.34 | 0.73 | 0 |
| Kahal et al. 2020 | PSG | 347 | -0.08 | -0.31 to 0.14 | 0.73 | 0.47 | 0.33 | 0 |
| Christ et al. 2024 | BQ | 77 | 0.11 | -0.36 to 0.58 | 0.47 | 0.64 | 0.62 | 0 |
| *Oestradiol* | | | | | | | | |
| Kahal et al. 2020 | PSG | 156 | -0.10 | -0.42 to 0.23 | 0.59 | 0.55 | NA | NA |
| Zhang et al. 2024 | PSG | 39 | 0.71 | 0.05 to 1.38 | 2.10 | 0.04* | NA | NA |
| Abbreviations: BQ, Berlin Questionnaire; CI, confidence interval; DHEAS, dehydroepiandrosterone sulfate; NA, not applicable; PSG, polysomnography; SHBG, sex hormone-binding globulin; SMD, standardized mean difference. Sensitivity analyses done by excluding one study at a time, and running the meta-analysis again. All analyses were done on inverse variance random-effects model. **p* values < .05 was considered significant. | | | | | | | | |

**Supporting Information 7**

***Studies that reported sex hormones &/or SHBG with OSA/SDB (data not suitable for meta-analysis)***

| **Author, year, country** | **Population, Setting** | **Study design** | **Study group** | **PCOS subgroup** | **Sex hormone** | **OSA or SDB** | **Findings in PCOS** |
| --- | --- | --- | --- | --- | --- | --- | --- |
| Fogel et al., 2001, USA | Adults, Community (controls) Hospital (cases) | Cross-sectional | PCOS  Non- PCOS | NA | Total T  Free T | OSA (AHI) by PSG | AHI correlated with serum total T (r = 0.52, *P* < .001) and Free T (r = 0.50, *P* < .05); Free T≥1.07 ng/dL: OR = 8.2 (1.3 to 49.7), *P* = .023. |
| Vgontzas et al. 2001, USA | Adults, General public (controls)  Sleep laboratory (cases) | Cross-sectional | PCOS  Non- PCOS | SDB  No SDB | Total T  Free T | SDB (AHI≥10 + clinical symptomatology) by PSG | No significant differences in total T or free T between PCOS (SDB vs. No SDB). |
| Yang et al., 2009, Taiwan | Adults, Community (control)  Obstetrics and gynaecology clinic in hospital (cases) | Cross-sectional | PCOS  Non- PCOS | NA | Total T  Androstenedione  SHBG | SDB (AHI_NREM_) by PSG | AHI_NREM_ correlated with T (B = 2.039, *P* = .032) and androstenedione (B = -1.036, *P* = .006), but not with SHBG. |
| Yang Rui et al., 2022, China | Adults (infertile), University hospital | Cross-sectional | PCOS | No OSA  OSA severity: Mild,  Moderate,  Severe | Total T  Androstenedione  Oestradiol | OSA (AHI≥5) by type III portable sleep monitor | T levels associated with OSA (OR = 1.11, *P* < .05). No difference in T and androstenedione levels between OSA vs. No OSA. Significant difference mild OSA vs. No OSA for oestradiol (Mean = 194.1, SD = 93.4 vs. Mean = 165.7, SD = 81.5 pmol/L, *P* < .05). |
| Tasali et al., 2006, USA | Adults, University endocrinology clinics, Sleep laboratory | Cross-sectional | PCOS | NA | Total T  Free T | SDB (AHI, ODI) by PSG | No significant correlations between total and free T and severity of SDB in women with PCOS. |
| Zhou et al., 2021, USA | Adults, PCOS clinic in university | Cross-sectional | PCOS | High-risk OSA  Low-risk OSA | Free T | OSA risk by BQ | High risk for OSA group had higher levels of free T compared with low risk OSA (*P* < .001). |
| Nandalike et al., 2011, USA | Adolescents,  Electronic medical information database | Cross-sectional | PCOS  Non-PCOS | SDB  No SDB | Total T  Free T | SDB by PSQ-SRDB | Total and free T levels similar between SDB vs. No SDB even after controlling for age, BMI and ethnicity. |
| Chatterjee et al. 2014 | Adults, Hospital clinic | Cross-sectional | PCOS | SDB  No SDB | Free T  SHBG | SDB (RDI≥5 + clinical symptoms or RDI>15),by PSG | No significant differences in free T (P = 0.167) and SHBG (P = 0.118) between PCOS (SDB vs. No SDB). |
| Tock et al., 2014, Brazil | Adults, Endocrinology division in university | Cross-sectional | PCOS | OSA  No OSA | Total T  Free T  DHEAS  androstenedione SHBG  Bioavailable T | OSA (AHI≥5) by PSG | After adjusting for BMI, hyperandrogenemia (free T >= 1.07 ng/dL) associated with OSA in women with PCOS (8.2 fold). SHBG lower in OSA vs. No OSA, *P* = .027. Free T higher in OSA vs. no OSA, *P* = .014. No significant difference between OSA vs. no OSA for androstenedione, DHEAS, total and bioavailable T. |
| Suri et al., 2016, India | Adults, Gynaecology outpatient department and endocrinology clinic in hospital | Cross-sectional | PCOS  Non-PCOS | SDB  No SDB | Free T | SDB (RDI≥5 + clinical symptoms or RDI>15) by PSG | Free T significantly correlated with RDI values (r = 0.377, *P* = .007). |
| Zhang et al. 2024, | Adults underwent IVF, University hospital | Prospective cohort | PCOS | OSA  No OSA | Total T | OSA (AHI≥5) by portable non-contact radar sleep monitor | No significant differences in Total T for PCOS (OSA vs. No OSA; P = 0.295). |
| Christ et al., 2024, USA | Adults,  University-based multidisciplinary PCOS clinic | Cross-sectional | PCOS | High-risk OSA  Low-risk OSA | Free T  Androstenedione  DHEAS  SHBG | OSA by BQ | Higher free T and lower SHBG (all *P* < .001) (High-risk OSA vs. Low-risk OSA). No significant differences between groups for androstenedione (*P* = .47) and DHEAS (*P* = .12). After adjusted for WHR and all reported variables, only SHBG remained significant in the multivariable model (B = -0.02 (0.01), *P* = .02). |
| Abbreviations: AHI, apnea-hypopnea index; BMI, body mass index; BQ, Berlin questionnaire; DHEAS, Dehydroepiandrosterone sulfate; OSA, obstructive sleep apnea; PCOS, polycystic ovary syndrome; PSG, polysomnography; PSQ-SRDB, Paediatric sleep questionnaire sleep-related disordered breathing; NA, not applicable; RDI, Respiratory disturbance index; SDB, Sleep disordered breathing; SHBG, sex hormone binding globulin; T, testosterone; WHR, waist-hip ratio. | | | | | | | |
|  | | | | | | | |

***Studies that reported sex hormones, SHBG, FAI &/or hirsutism with sleep disturbances (data not suitable for meta-analysis)***

| **Author, year, country** | **Population, Setting** | **Study design** | **Study group** | **PCOS subgroup** | **Sex hormone** | **Sleep disturbances** | **Findings in PCOS** |
| --- | --- | --- | --- | --- | --- | --- | --- |
| Caltekin et al., 2021, Turkey | Adults, Gynaecology and neurology outpatient clinics at hospital | Case-control | PCOS  Non-PCOS | Poor sleep quality  Normal sleep quality  EDS  No EDS  RLS  No RLS | Total T | Sleep quality by PSQI  Daytime sleepiness by ESS  RLS by IRLSSG criteria | No significant correlations between T levels and PSQI and ESS. No significant difference in T levels RLS vs No RLS. |
| Karasu et al., 2021, Turkey | Adults, Gynaecology outpatient clinic at university | Cross-sectional | PCOS  Non-PCOS | NA | Free T  Hirsutism from mFG score ≥8 | Sleep quality by PSQI | No significant correlations between free T, mFG scores and PSQI score. Hirsutism showed significantly longer sleep latency compared to those without hirsutism, but no numeric data was reported. |
| Shreeve et al., 2013, UK | Adults, Gynaecology outpatient clinic in hospital | Case-control | PCOS  Non-PCOS | Poor sleep quality  Normal sleep quality | Total T  FAI  SHBG  Oestradiol | Sleep quality by PSQI | No significant correlations between sleep quality and Total T, FAI, SHBG and oestradiol. |
| Turan et al., 2022, Turkey | Adults, Gynaecology and obstetrics outpatient clinic | Case-control | PCOS  Non-PCOS | Poor sleep quality  Normal sleep quality | Total T  DHEAS | Sleep quality by PSQI | After controlling for age and BMI, T and DHEAS levels were not significantly associated with PSQI. |
| Nandalike et al., 2011, USA | Adolescents,  Electronic medical information database | Cross-sectional | PCOS  Non-PCOS | EDS  No EDS | Total T  Free T | Daytime sleepiness by ESS | T levels similar EDS vs. No EDS even after controlling for age, BMI and ethnicity. |
| Hachul et al., 2019, Brazil | Adults, Endocrinology division in university | Cross-sectional | PCOS  Non-PCOS | NA | Total T Clinical/biochemical HA | Sleep quality parameters by PSG  Daytime sleepiness by ESS  Sleep quality by PSQI | After controlling for age, no differences in ESS, PSQI scores and PSG-sleep wake parameters (i.e., sleep efficiency, sleep latency, wake after sleep onset and number of AHI events) between HA vs. without HA. |
| de Sousa et al., 2011, Germany | Adolescents, Outpatient obesity and endocrine department hospital | Cross-sectional | PCOS  Non-PCOS | NA | Total T  DHEAS androstenedione | Sleep apnea/quality parameters by PSG | No significant correlations between PSG variables and androgen levels. |
| Abbreviations: BMI, body mass index; DHEAS, Dehydroepiandrosterone sulfate; EDS, Excessive daytime sleepiness; ESS, Epworth sleepiness scale; FAI, free androgen index; mFG, modified Ferriman-Gallwey; PCOS, polycystic ovary syndrome; PSG, polysomnography; PSQI, Pittsburgh sleep quality index; NA, not applicable; SHBG, sex hormone binding globulin; T, testosterone. | | | | | | | |
